# Dynamic protein interaction modules in human hepatocellular carcinoma progression

**DOI:** 10.1186/1752-0509-7-S5-S2

**Published:** 2013-12-09

**Authors:** Hui Yu, Chen-Ching Lin, Yuan-Yuan Li, Zhongming Zhao

**Affiliations:** 1 Department of Biomedical Informatics, Vanderbilt University School of Medicine, Nashville, TN 37203, USA; 2 Shanghai Center for Bioinformation Technology, Shanghai 201203, P. R. China; 3 Department of Psychiatry, Vanderbilt University School of Medicine, Nashville, TN 37212, USA; 4 Department of Cancer Biology, Vanderbilt University School of Medicine, Nashville, TN 37232, USA; 5 Center for Quantitative Sciences, Vanderbilt University School of Medicine, Nashville, TN 37232, USA

## Abstract

**Background:**

Gene expression profiles have been frequently integrated with the human protein interactome to uncover functional modules under specific conditions like disease state. Beyond traditional differential expression analysis, differential co-expression analysis has emerged as a robust approach to reveal condition-specific network modules, with successful applications in a few human disease studies. Hepatocellular carcinoma (HCC), which is often interrelated with the *Hepatitis C *virus, typically develops through multiple stages. A comprehensive investigation of HCC progression-specific differential co-expression modules may advance our understanding of HCC's pathophysiological mechanisms.

**Results:**

Compared with differentially expressed genes, differentially co-expressed genes were found more likely enriched with *Hepatitis C *virus binding proteins and cancer-mutated genes, and they were clustered more densely in the human reference protein interaction network. These observations indicated that a differential co-expression approach could outperform the standard differential expression network analysis in searching for disease-related modules. We then proposed a differential co-expression network approach to uncover network modules involved in HCC development. Specifically, we discovered subnetworks that enriched differentially co-expressed gene pairs in each HCC transition stage, and further resolved modules with coherent co-expression change patterns over all HCC developmental stages. Our identified network modules were enriched with HCC-related genes and implicated in cancer-related biological functions. In particular, *APC *and *YWHAZ *were highlighted as two most remarkable genes in the network modules, and their dynamic interaction partnership was resolved in HCC development.

**Conclusions:**

We demonstrated that integration of differential co-expression with the protein interactome could outperform the traditional differential expression approach in discovering network modules of human diseases. In our application of this approach to HCC's gene expression data, we successfully identified subnetworks with marked differential co-expression in individual HCC stage transitions and network modules with coherent co-expression change patterns over all HCC developmental stages. Our results shed light on subtle HCC mechanisms, including temporal activation and dismissal of pivotal functions and dynamic interaction partnerships of key genes.

## Background

With great improvement in both the quantity and quality of human protein-protein interaction data, a comprehensive human protein interaction network was created and serves as the backbone of many human disease studies [1-4]. However, the reference protein interaction network masks the *in vivo *spatial and temporal contexts and unrealistically integrates all *in vitro *molecular interactions together. Therefore, it is imperative to identify condition-specific protein interaction network modules that have more spatiotemporal biological relevance to disease studies. Recently, in response to the call for dynamic interactomes [5,6], many efforts have been made to extract active subnetworks by integrating stage-wise or time series gene expression data into protein interaction network [7-20].

As the most intuitive and straightforward expression feature, differential expression statistics are often overlaid onto protein interaction network, and subnetworks enriched for differential expression genes are fetched [7-11]. However, since protein interaction network is essentially a model of relations among biological molecules, a more precise characterization of dynamic protein interaction network would result from addressing the relation changes than the entity changes. The differential co-expression analysis, which investigates the changes of expression correlations between genes, has arisen as a promising alternative to traditional differential expression analysis [21]. While differential co-expression is related to co-expression, the gene-gene co-expression in a comparative condition should not be a prerequisite [22,23]. In relation to protein interaction network studies, however, co-expression analyses were more frequently performed than differential co-expression analyses [12-16]. Recently, the use of differential co-expression analyses to uncover dynamic protein interaction network modules specific to human diseases has begun [17-20]. These studies have made important discoveries in heart failure [18], glioma prognosis [17], HIV infection [19], and other diseases. However, some of these studies were predisposed to co-expression analysis, which may have limited the potential of their approaches.

Hepatocellular carcinoma (HCC) is the most common type of liver cancer, and it is often interrelated with the *Hepatitis C *virus (HCV). A typical HCC progression may go through the following successive stages: Normal (N), Cirrhosis (C), Dysplasia (D), Early HCC (E), and Advanced HCC (A). As a gradually-developed carcinoma with marked pre-neoplastic stages and neoplastic stages, HCC calls for a better understanding at the genomic level of the origin and transitions of its carcinogenesis. A well-designed, multi-stage HCC expression dataset has been analyzed by different groups from the perspective of differential expression with [24] or without network context [25], but the dataset has not been explored for differential co-expression yet. Like the successful attempt in a multi-state human colorectal cancer study [20], a differential co-expression analysis of protein interaction network modules may advance our understanding of HCC's pathophysiological mechanisms.

**Figure 1:**
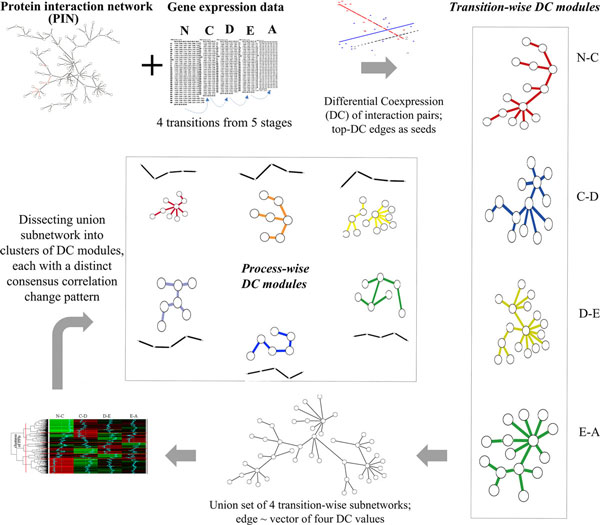
**Differential co-expression analysis of protein interaction network for human hepatocellular carcinoma (HCC) progression**. Four arrows in this figure indicate major analysis flow. Arrow 1: for each protein interaction pair (edge), an expression correlation value was calculated for each of five HCC stages, and a differential correlation value (*dC*) was calculated for each of four stage transitions. Edges with the highest *dC *values were used as seeds in a search of a differential co-expression subnetwork. Four subnetworks were retrieved from the PIN for the four HCC stage transitions, respectively. Arrow 2: the four transition-wise subnetworks were combined into a union set, in which each edge was associated with four *dC *values. Arrow 3: the edges in the union subnetwork were clustered based on similarity in those four-*dC *data vectors. Arrow 4: six clusters of differential co-expression protein-interaction modules were determined, each characterized with a distinct, coherent co-expression change pattern over the whole HCC process.

In this study, we first comparatively evaluated Differentially Expressed Genes (DEGs) and Differentially Co-expressed gene Pairs (DCPs) for their qualification for subnetwork seeds, and as a result proved the improved validity of searching for protein interaction subnetworks from seeds of DCPs than DEGs. We then proposed a differential co-expression network approach to uncover gene modules involved in HCC development. Specifically, we identified subnetworks that enriched DCPs in each HCC transition stage, and further resolved modules with coherent co-expression change patterns over all HCC developmental stages (Figure 1). Our identified network modules were found to be enriched with HCC-related genes and implicated in cancer-related biological functions. The results shed light on subtle HCC mechanisms, including temporal activation and dismissal of pivotal functions and dynamic interaction partnerships of key genes.

## Results

### Differential co-expression protein interactions as seeds in subnetwork searches

The stage-wise Pearson correlation coefficient (*r*) values of gene expression profiles were calculated for all possible pairs formed by the genes in the reference protein interaction network. For each HCC stage, we compared all pairs' Pearson correlation coefficients (absolute values) with those of the protein interaction pairs' subset (absolute values) using the two-sample Kolmogorov-Smirnov test. We found that at all stages the protein interaction pairs' absolute Pearson correlation coefficients were larger than the total control at a significant level (*p *< 0.001 for N, C, and D) or marginally significant level (*p *< 0.1, for E and A).

Then, we derived the differential correlation values (*dC*) for each protein interaction pair and each stage transition. In this manner, each protein interaction pair was described with a vector of four *dC *values. A sizeable portion of distant protein interaction pairs characterized with noticeably larger *dC *values were discovered in an outlier analysis of the *dC *data matrix (Additional file 1).

Most approaches to extracting protein interaction subnetworks, including our previous one [24], were seeded from a set of DEGs. In this work, we intended to use DCPs as alternative seeds; therefore, we initially set out to investigate if DCPs were a better option for seeds. Successively inclusive sets of top-DEGs (based on absolute log fold changes of mean expression) or top-DCPs (based on absolute *dC *values) were chosen at three increasing levels: 0.1%, 0.5%, or 1%. For comparability, we derived corresponding sets of "Differentially Co-expressed Genes" (DCGs) as the genes involved in DCPs and compared the derived DCGs with the DEGs at the same levels.

First, an ideal seed gene set should be related to the studied subject, which in our case was the HCV-induced HCC progression. For this criterion, we evaluated top-DEGs and top-DCGs in terms of their enrichment of HCV-protein-binding (HCB) proteins, cancer-mutated genes from Cancer Gene Consensus (CGC), or HCC-responsive genes (HCR). As clearly shown in Table 1 HCV-binding proteins were more enriched in top-DCGs than in top-DEGs: except for one case, all top-DCG sets were significantly enriched with HCV-binding proteins, whereas none of top-DEG sets were enriched with HCV-binding proteins. In terms of HCC-responsive genes and cancer-mutated genes, advantages were still existent using DCGs compared to DEGs (Additional file 1).

**Table 1:** Proportion of *Hepatitis C *viru**s**-binding proteins in top ranked gene seeds

Top level^a^	Gene set	HCC transition^b^			
		
		N-C	C-D	D-E	E-A
0.1%	DEG	0/11	1/11	0/11	1/11
	DCG	7/63*	9/65***	10/60***	4/63
0.5%	DEG	3/55	2/55	2/55	4/55
	DCG	25/290***	36/279***	27/287***	27/288***
1.0%	DEG	6/110	3/110	5/110	6/110
	DCG	47/524***	51/538***	36/523***	41/559***

In each cell, the proportion is shown as ″x/y″, with y for top-ranked genes and × for genes of interest. Enrichment *p*-value < 0.01 (*) or < 0.0001 (***) are marked.

^a^Top-level is the fraction of the top-ranked genes/pairs in the total network nodes/edges. For differential expression genes (DEG), we used the node-wise fraction; for differential co-expression genes (DCGs) deriving from pairs, we used the edge-wise fraction. A same fraction of pairs involve a greater number of genes.

^b^Transition names: Normal to Cirrhosis (N-C), Cirrhosis to Dysplasia (C-D), Dysplasia to Early HCC (D-E), and Early HCC to Advanced HCC (E-A).

Additionally, as many subnetwork-searching algorithms (e.g. Steiner minimum tree [26]) implicitly assume, a set of seed genes should ideally cluster densely in the whole network so that they can be connected into a subnetwork via a limited number of mediators. It therefore follows that a set of seed genes should have an average pairwise distance shorter than that between random pairs. As a baseline, the shortest paths in the whole protein interaction network were 3.81 ± 0.86 (any disconnected protein pairs that were unable to be connected via any path were excluded in this calculation). We found that the shortest paths among DCG seeds were generally one-step shorter than random pairs, while those among DEG seeds were generally very close to random pairs (Table 2). In fact, the advantage of DCG seeds over DEG seeds was underestimated here, as disconnected pairs were found in DEG sets but not in DCG sets (footnotes of Table 2). In conclusion, the seed DCGs were clustered more densely in protein interaction network than the seed DEGs, implying a more likely formation of compact, expression-coherent subnetworks when using seed DCPs than seed DEGs.

**Table 2:** Average shortest length among top ranked genes

Top level	Gene set	HCC transition			
		
		N-C	C-D	D-E	E-A
0.1%	DEG	3.61 ± 0.59	3.25 ± 0.86	3.40 ± 0.91	4.25 ± 0.86
	DCG	2.96 ± 0.71	2.94 ± 0.74	2.72 ± 0.75	2.98 ± 0.75
0.5%	DEG	3.70 ± 0.78 ^**a**^	3.62 ± 0.85	3.76 ± 0.82	3.62 ± 0.85 ^**b**^
	DCG	2.89 ± 0.71	2.81 ± 0.68	2.82 ± 0.73	2.89 ± 0.70
1.0%	DEG	3.60 ± 0.81 ^**c**^	3.63 ± 0.84	3.67 ± 0.81 ^**d**^	3.60 ± 0.87 ^**e**^
	DCG	2.87 ± 0.69	2.85 ± 0.76	2.86 ± 0.70	2.90 ± 0.71

There were 3.6% ^**a**^, 10.7% ^**b**^, 1.8%**^c^**, or 5.4%**^d,e ^**disconnected DEG pairs and they were excluded from the average shortest length calculation.

Therefore, the relative advantage of DCPs as seeds in protein interaction subnetwork searches was demonstrated against traditional DEGs, which justified our subsequent edge-wise subnetwork searches from these top-ranked seed DCPs.

### Differential co-expression subnetwork in each HCC stage-transition

In order to avoid oversized subnetworks, we used the top 0.1% DCPs (65 DCPs) as seeds in the subnetwork searches (see Materials and methods). These top 65 DCPs involved 123, 124, 118, and 123 seed genes in the four stage transitions, respectively (Additional file 2).

One subnetwork was retrieved for each transition between consecutive HCC stages, and the four subnetworks are termed "transition-wise" subnetworks hereafter. The properties of these transition-wise subnetworks are summarized in Table 3 and Additional file 1, and full subnetworks can be reviewed in Additional files 3 and 4. While the overall edge-to-node ratio in protein interaction network is about 5.9 (64,865 to 10,953), the same statistics in the subnetworks is about 1, reflecting a selective recruitment of edges into the subnetworks. The *r *values and *dC *values associated with links in the subnetworks are more conspicuous than the background level in protein interaction network (Additional file 1), indicating an effective condensation of differential co-expression relations.

**Table 3:** Transition-wise differential co-expression protein interaction subnetworks

Transition	# nodes	# edges	**HCC-related genes **^**a**^			**Hub genes **^b^
				
			HCB	CGC	HCR	
N-C	307	310	28***	29***	154*	ARRB1, **APC**, **CDKN2A**, *CSNK2A1*, CSNK2A2, ***EP300***, ESR1, *GRB2*, *HCK*, MCC, ***PIK3R1***, PPP1CC, SRRM2, SUMO2, TSC22D1, YWHAG, *YWHAZ*
C-D	102	103	13***	10*	44	**APC**, GFI1B, NEDD4, UBE2D3
D-E	104	103	10*	13***	58*	*IKBKE, PIK3R1, YWHAZ*
E-A	103	102	9*	11*	60*	**EGFR**, *FYN*, IKBKG, *PRMT1*,YWHAG

^a^See Materials and methods for explanation of the three HCC-related gene sets. Significance levels of HCC-related gene enrichment are labeled by *(*p *< 0.01) and ***(*p *< 0.0001).

^b^Hubs are genes with six or more connected neighbors. *Hepatitis C *virus -binding genes (*italicized*) and cancer-mutated genes (**bold**) are marked.

The coverage of HCC-related genes in the transition-wise subnetworks was studied to evaluate the relevance of our subnetworks in relation to HCC development. Three types of HCC-related genes, HCB, CGC, and HCR, were examined separately. Almost all four transition-wise subnetworks were enriched with these HCC-related genes (Table 3). Then, following the example of our previous work [24], we defined proteins with more than six connections as hubs and obtained a total of 25 hubs in the four subnetworks (Table 3). Among the 18 Gene Ontology (GO) [27] biological processes terms enriched within these hub genes (Additional file 1), some are evidently related to HCC pathogenesis, such as "interspecies interaction between organisms", "immune response-activating signal transduction" [28], and "platelet activation" [29]. In summary, nine hubs are targeted by HCV proteins, and five are mutated in cancer. Of the only five liver-cancer-associated genes from CGC, APC [30] appeared as a recurrent hub in multiple subnetworks. These observations suggested that our transition-wise subnetworks are highly relevant to the development of HCC.

**Figure 2:**
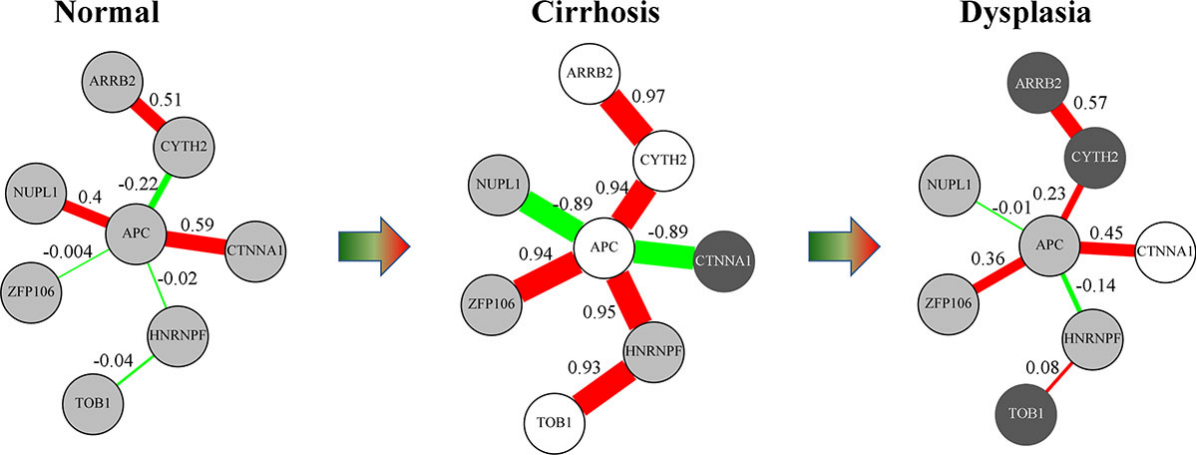
**APC-centered protein interaction module characteristic for dynamic co-expression patterns over hepatocellular carcinoma precancerous stages**. Edge widths are proportional to the expression correlation values (edge weights). Red edge: positive expression correlation; green edge: negative expression correlation. Light gray node: non-differential expression; dark gray node: up-regulation (t-test, *p *< 0.05); white node: down-regulation (t-test, *p *< 0.05).

Then, we investigated the overlapping genes and edges between transition-wise subnetworks. There was a moderate overlap in subnetwork nodes (as high as 28.4%) but a minor overlap in subnetwork edges (as high as 13.6%). Altogether, we observed 16 differentially co-expressed protein interaction pairs recurrent in multiple subnetworks, two of which involved N-C and E-A transitions, and the other 14 of which involved consecutive N-C and C-D transitions (Additional file 1). For all 14 N-C-D continuous-changing protein interaction pairs, the expression correlation values reached a significantly high level in cirrhosis but not in normal or dysplasia stages (FDR threshold of 0.25, equivalent to |*r*| > 0.76 in cirrhosis). Remarkably, seven of these 14 protein interaction pairs were connected into a APC-centered module (Figure 2), in which four proteins, APC, CYTH2, ARRB2, and CTNNA1, were involved in the "Signaling events mediated by Hepatocyte Growth Factor Receptor (c-Met)" [31]. Aside from the important core protein APC, another protein CTNNA1 may be worth special attention as well, as it takes part in the E-cadherin/catenin complex whose abrogation was implicated in the carcinogenesis of several malignancies [32]. The aggregation of differential co-expression relations around the HCC-mutated gene *APC *and the involvement of quite a few HCC-related genes suggest that the APC-centered protein interaction module (Figure 2) may encode pivotal HCC-pathogenesis mechanisms for which further investigation is warranted.

### Differential co-expression modules in HCC progression process

**Figure 3:**
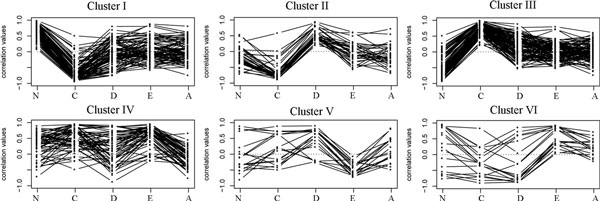
**Expression correlation change patterns of six clusters of differential co-expression protein interaction modules**. Abscissa includes five Hepatocellular carcinoma (HCC) stages: Normal (N), Cirrhosis (C), Dysplasia (D), E (Early HCC), and A (Advanced HCC).

While the transition-wise analysis reveals differential co-expression subnetworks that are remarkable in single stage transitions, a process-wise analysis may catalogue protein interaction network modules based on their co-expression change patterns over all HCC development stages. Therefore, we obtained the union of the four transition-wise subnetworks and dissected them into clusters of modules by clustering the vectors of *dC *values associated with each protein interaction pair (Euclidean distance measure; complete linkage clustering). This approach resulted in six clusters of protein interaction pairs with mutually distinct correlation change patterns (Figure 3), where each cluster was comprised of multiple disconnected network modules (Table 4 and Additional files 5 and 6). Further investigation revealed the dynamic correlation change patterns of each cluster (Figure 3). Clusters I, II, and III were more dynamic in early, precancerous phases (N-C-D), while clusters IV, V, and VI were more dynamic in later, cancerous phases (D-E-A).

**Table 4:** Six clusters of process-wise differential co-expression protein interaction modules

Cluster ID	# node	# edge	# components (size ≥5)	Size of largest component	# HCV-binding genes
I	96	87	10	13	6
II	40	36	4	20	9***
III	122	112	10	21	14***
IV	60	56	4	19	8 *
V	21	18	3	7	3 *
VI	20	18	2	13	2

*Hepatitis C *virus (HCV)-binding protein enrichment significance levels: ^* ^for *p *< 0.01 and ^*** ^for *p *< 0.0001.

A functional enrichment analysis was performed to uncover the biological themes of each cluster (see Materials and methods), and the results are summarized in Table 5. In coherence with HCV-associated HCC progression, some relevant biological processes were discovered, such as "viral reproduction" (cluster I), "interspecies interaction between organisms" (cluster III and cluster IV), and "wound healing" (cluster V) [33].

**Table 5:** Gene Ontology (GO) Biological Processes enriched in clusters of differential co-expression protein interaction modules

Cluster	GOID	Term	# expec-ted genes	# genes	Adjusted *p*-value *
I	GO:0071842	cellular component organization at cellular level	23.3	44	0.0002
I	GO:0007346	regulation of mitotic cell cycle	2.6	12	0.0004
I	GO:2000241	regulation of reproductive process	1.2	8	0.0006
I	GO:0009968	negative regulation of signal transduction	4.3	15	0.0006
I	GO:0043066	negative regulation of apoptotic process	4.9	16	0.0006
I	GO:0031577	spindle checkpoint	0.4	5	0.0007
I	GO:0032088	negative regulation of NF-kappaB transcription factor activity	0.4	5	0.0007
I	GO:0000086	G2/M transition of mitotic cell cycle	1.3	8	0.0007
I	GO:0048522	positive regulation of cellular process	23.2	41	0.0007
I	GO:0006366	transcription from RNA polymerase II promoter	10.2	24	0.0007
I	GO:0042221	response to chemical stimulus	20.2	37	0.0008
I	GO:0031623	receptor internalization	0.4	5	0.0008
I	GO:0048468	cell development	10.5	24	0.0008
I	GO:0050658	RNA transport	1.0	7	0.0008
I	GO:0016032	viral reproduction	3.7	13	0.0009
II	GO:0043065	positive regulation of apoptotic process	2.0	11	0.0003
II	GO:0006366	transcription from RNA polymerase II promoter	4.3	15	0.0005
III	GO:0016567	protein ubiquitination	4.5	17	0.0001
III	GO:0000075	cell cycle checkpoint	2.7	13	0.0001
III	GO:0042981	regulation of apoptotic process	12.9	30	0.0001
III	GO:0000165	MAPK cascade	4.4	16	0.0001
III	GO:0032268	regulation of cellular protein metabolic process	12.6	29	0.0001
III	GO:0010627	regulation of intracellular protein kinase cascade	5.7	18	0.0002
III	GO:0050863	regulation of T cell activation	2.4	11	0.0002
III	GO:0007346	regulation of mitotic cell cycle	3.3	13	0.0002
III	GO:0044419	interspecies interaction between organisms	4.4	15	0.0003
III	GO:0006511	ubiquitin-dependent protein catabolic process	4.0	14	0.0003
III	GO:0045892	negative regulation of transcription, DNA-dependent	7.7	20	0.0005
III	GO:0007265	Ras protein signal transduction	2.8	11	0.0006
III	GO:0007173	epidermal growth factor receptor signaling pathway	1.9	9	0.0007
III	GO:0051090	regulation of sequence-specific DNA binding transcription factor activity	3.3	12	0.0007
III	GO:0009967	positive regulation of signal transduction	6.7	18	0.0007
III	GO:0016310	phosphorylation	13.6	28	0.0008
III	GO:0045732	positive regulation of protein catabolic process	0.8	6	0.0008
III	GO:0030518	intracellular steroid hormone receptor signaling pathway	1.1	7	0.0008
III	GO:0042770	signal transduction in response to DNA damage	1.5	8	0.0009
III	GO:0080134	regulation of response to stress	6.9	18	0.0009
III	GO:0008285	negative regulation of cell proliferation	5.1	15	0.0009
IV*	GO:0044419 *	interspecies interaction between organisms	2.1	11	0.0020
IV*	GO:0000904 *	cell morphogenesis involved in differentiation	3.3	12	0.0099
IV*	GO:0007165 *	signal transduction	18.8	33	0.0099
V	GO:0065008	regulation of biological quality	4.1	13	0.0008
V	GO:0001775	cell activation	1.4	8	0.0009
V	GO:0018193	peptidyl-amino acid modification	1.0	7	0.0009
V	GO:0048011	nerve growth factor receptor signaling pathway	0.5	5	0.0009
V	GO:0043066	negative regulation of apoptotic process	1.1	7	0.0009
V	GO:0000165	MAPK cascade	0.8	6	0.0009
V	GO:0042060	wound healing	1.2	7	0.0009
V	GO:0045860	positive regulation of protein kinase activity	0.8	6	0.0009
V	GO:0071375	cellular response to peptide hormone stimulus	0.5	5	0.0009

*Threshold value for adjusted *p*-value is 0.01 (Cluster IV) or 0.001 (clusters I, II, III, and V). No GO term was found to be enriched for cluster VI with either threshold value.

Among the early-active clusters, cluster I and cluster III are representatives of two opposite trends: when the disease progresses from the normal stage through cirrhosis to dysplasia, Pearson correlation coefficient values in cluster III go upward and then downward, while those in cluster I show a trend that is exactly reversed. Interestingly, some enriched functions of these two clusters happen to be contrary to each other (Table 5). For instance, "negative regulation of apoptotic process" is enriched in cluster I, while "negative regulation of cell proliferation" is enriched in cluster III. It seems that, at the precancerous stages of HCC, the cells are coordinating some proliferation-inhibiting genes while simultaneously dismissing some apoptosis-inhibiting genes. These functions are possibly spontaneous calibration mechanisms taking place in precancerous stages to "halt" the potential carcinogenesis. As another probable calibration action, expression coordination is enhanced in "positive regulation of apoptotic process" (cluster II) at a later precancerous stage, dysplasia.

Of the three later-activated clusters, cluster V, where protein interaction pairs undergo consistent correlation enhancements from early HCC to advanced HCC (Figure 3), is enriched with the greatest number of functional terms (Table 5). Seven genes in cluster V are involved in "negative regulation of apoptotic process", and their enhanced correlations in the advanced HCC samples likely indicate an ultimate breakdown of the apoptosis program. Other potentially relevant terms tagged to cluster V include "wound healing" and "MAPK cascade," which are functions frequently implicated in carcinogenesis or cancer metastasis [33,34]. Cluster IV, a group of protein interaction pairs with collapsed correlations in advanced HCC, is enriched with "interspecies interaction between organisms."

Interestingly, 27 interfacing proteins were found interacting with partners from different dynamic co-expression clusters, which contain both highly-connected proteins and lowly connected proteins. Ten proteins (YWHAZ, TSC22D1, APC, YWHAG, IKBKG, ARRB1, ESR1, FYN, GRB2, and NEDD4, ordered by their connection degrees decreasingly) are specifically noteworthy because they are the top 10 strongest-connected proteins in the process-wise subnetwork (the union of the six clusters of protein interaction modules) and include the top 3 proteins with the highest betweenness as well (APC, YWHAZ, and IKBKG). Lowly-connected interfacing proteins are no less interesting, as they include the HCV-binding protein SMURF2 and the cancer-mutated gene *TPR*, both of which have a connectivity of three. In all, many of these interfacing proteins are potentially related to HCC, as 17 are covered in the HCC-responsive gene sets and five are targeted by HCV proteins (Additional file 1).

**Figure 4:**
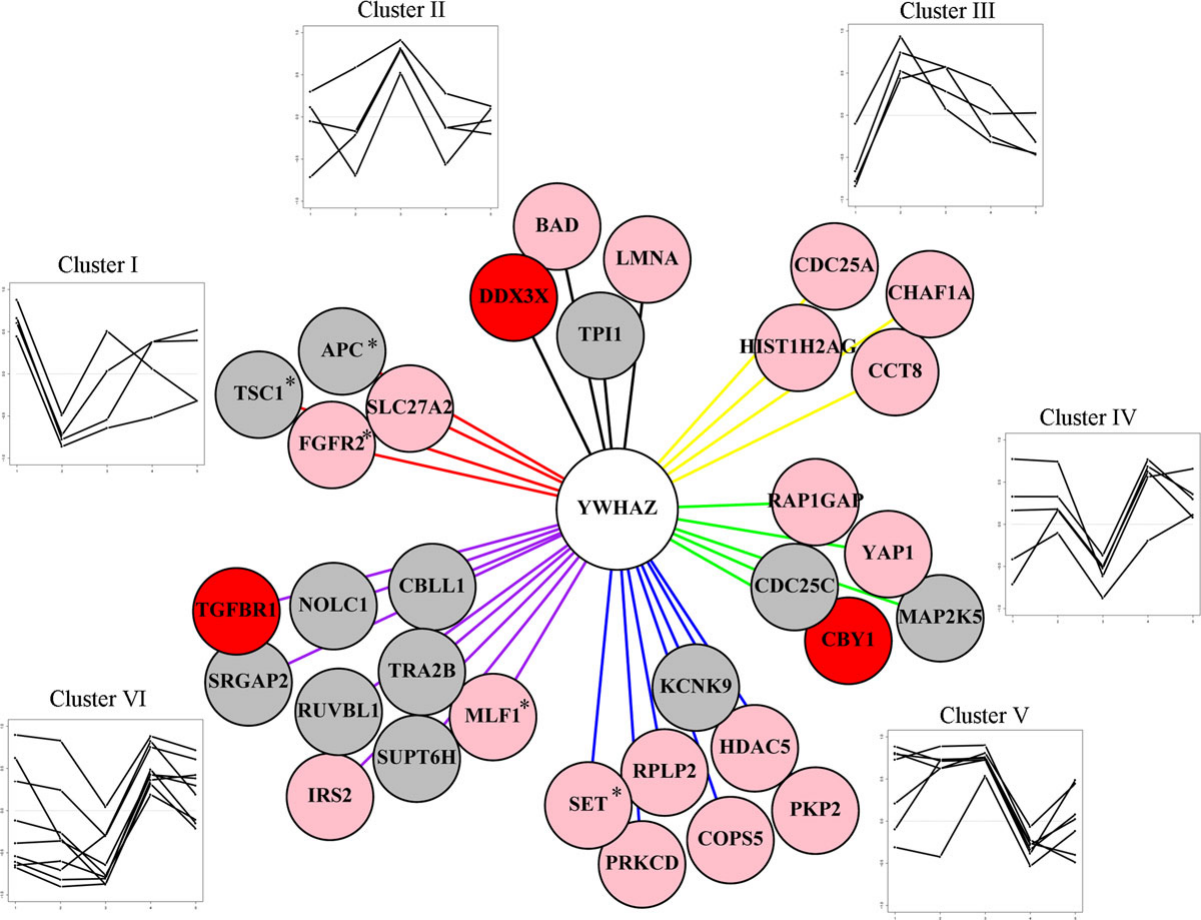
**Thirty-three interactions of YWHAZ were categorized into six clusters based on their dynamic co-transcription profiles**. *Hepatitis C *virus protein-binding genes (in red), hepatocellular carcinoma-responsive genes (in pink), and cancer-mutated genes (*) were marked.

Of all 27 interfacing proteins, YWHAZ is the only one that interfaces with all six clusters of protein interaction modules (Figure 4). YWHAZ is a HCV-binding protein and is covered in two HCC-responsive gene sets (Additional file 1). Additionally, it has been implicated in HCC in terms of copy number alteration [35] and drug-responsive differential expression [36,37]. We extracted all protein interaction pairs (edges) connected to YWHAZ in the process-wise subnetwork and separated them into the six characteristic clusters (Figure 4). This YWHAZ-centered module contains four cancer-mutated genes, three HCV-binding proteins, and numerous HCC-responsive differential expression genes (Figure 4). Interestingly, three cancer-mutated genes had a similar differential co-expression pattern with YWHAZ, and all have a sharp correlation collapse in cirrhosis (Figure 4, cluster I). Although further investigation is warranted, the dynamic dissection of YWHAZ's interaction partners in this work provide unique clues to subtle mechanisms of HCC pathophysiology.

### Verification of the approach in an independent hepatocellular carcinoma dataset

An independent gene expression dataset, GSE14323, was used to verify the N-C subnetwork. We observed that overall there was no correlation between the *dC *values of the 56,142 overlapping protein interaction pairs in GSE6467 and GSE14323 (*r *< 0.01). Notably, the *dC *values of the top 0.1% DCPs in GSE6467 (seed DCPs) are significantly positively correlated with those in GSE14323 (*r *= 0.23, *p *= 0.04). A similar significant positive correlation was observed for the 286 N-C subnetwork protein interaction pairs between GSE6467 and GSE14323 (*r *= 0.10, *p *= 0.04).

We then performed an analogous subnetwork search using GSE14323. Starting from top 1% DCPs, we obtained a subnetwork with 255 links formed by 280 unique genes. There are 34 genes shared by the two N-C subnetworks, including one common hub - APC. FYN, a hub discovered in GSE14323 N-C subnetwork, is also a hub in E-A subnetwork of GSE6467. The GSE14323 subnetwork harbors an additional hub, CTNNB1, which is a confirmed HCC-mutated gene [30]. Here, the recurrence of HCC-related hub genes from independent datasets indicates the validity of our differential co-expression network approach.

## Discussion

In this work, we integrated differential co-expression analysis with protein interaction network and applied a network-based approach to uncover HCC-specific dynamic protein interaction modules. Our framework has generated a valuable set of plausibly HCC-implicated genes and protein interaction pairs for follow-up investigations. Currently, the number of assured HCC-implicated genes remains very small. In Cancer Gene Census [38], there are only five genes explicitly associated with liver cancer. Among these five genes, *APC *appears as a recurrent hub in our subnetworks (N-C and C-D), and it was also verified as a hub in the N-C subnetwork using another independent dataset. In addition, some plausibly implicated genes stand out in our modules. These genes include those mutated in other cancer types and bound with HCV proteins (e.g. PIK3R1 and EP300*)*, genes interfacing with alternative partner groups (e.g. *YWHAZ*), and genes manifesting both differential co-expression and differential expression (e.g. *ESR1*). Some genes meet multiple prioritization criteria; for instance, *EEF1D *is a DEG involved in a DCP and it interfaces with alternative partner groups. In summary, genes highlighted in our dynamic modules may serve as a set of practically plausible candidate targets for follow-up HCC studies.

Moreover, our framework provides a way to reveal protein interaction rewiring during HCC progression. For instance, alternative interaction partners were activated for the proteins shared in the N-C and C-D subnetworks. In our focused APC*-*centered module (Figure 2), two distinct groups of APC partners were distinguished by discriminating their correlation relationships with APC in the N-C-D progression: one group, consisting of CTNNA1 and NUPL1, demonstrated a NS-HN-NS (NS: non-significance; HN: High-Negative) correlation pattern with APC, while the other group, consisting of ZFP106, CYTH2, and HNRNPF, displayed an opposite NS-HP-NS (HP: High-Positive) pattern. Such alternative partnerships in company with condition changes could be actual instances of the "date hubs" conceptualized several years ago [39].

Comparing our differential co-expression subnetworks with previous differential expression subnetworks [24], we observed a significant number (21, hyper-geometric test, *p *< 0.001) of common genes if ignoring transition-to-transition mapping. This result indicates that a significant number of genes were remarkable in both differential co-expression and also differential expression, yet the two types of expression changes may not happen simultaneously. Interestingly, most of these shared genes manifested an earlier differential co-expression and then a later differential expression (Additional file 1). A similar trend was seen with the three genes overlapping the original differential expression study [25] (Additional file 1). This observation suggested that differential co-expression is a more upstream event than differential expression in biological systems. Along the central dogma, a causal mutation at the genetic level is unambiguously the most upstream event. Such a causal mutation is transduced through a conceptual biological information flow and ultimately results in consequences at the molecular, cellular, and bodily level. Closely succeeding the initial mutation event is transcription dysregulation, which sometimes manifests itself as "altered relationships" between regulators and targets or among targets. Differential expression, as the molecular-level output of the information flow, can occur with the causal regulator's direct targets as well as indirect targets. While a certain time-lag was necessary for a microRNA's regulatory effects to propagate fully to the secondary targets [40], it is likely that, at a higher-level and a larger scale, genes' differential expression phenomena may lag behind their differential co-expression circumstances by a time-phase that corresponds to one or more disease stages. This putative precedence of differential co-expression over differential expression deserves a systematic investigation in extended progressive disease datasets.

With the stage-wise HCC expression data, the proposed differential co-expression network approach resolved modules with coherent co-expression change patterns over all HCC developmental stages and even deciphered the temporal activation/dismissal of involved functions. In our results, negative regulation of the apoptotic process was found to be dismissed at early precancerous phases but was recruited in established HCC; positive regulation of the apoptotic process was to be coordinated in precancerous phase dysplasia. These mutually consistent functional dynamics may suggest broad, coordinated anti-cancer calibration mechanisms taking place in precancerous stages. Relating to the putative precedence of differential co-expression over differential expression, these functional dynamics may underpin pivotal HCC stage transitions for which more elaborate studies are warranted towards a potential early phase HCC intervention.

From a methodological point of view, our approach has some similarity to a few previous studies [18,20,41]. Like us, all of these studies have integrated protein interaction network with expression data, and their outputs have included sort of protein interaction subnetworks/modules. The most striking difference between our approach and the cited ones is that we first quantified differential co-expression of each protein interaction pair (edge) and then set out to search for the organization of those differentially co-expressed edges. Lin et al. [18] and Chuang et al. [20] were similar to each other in that they first constructed a co-expression protein interaction network for each disease state and then compared the two obtained network in terms of topological features or functional annotations; Gu et al. [41] devised a unique clustering framework which has taken into account both protein interaction network topology and gene expression correlation. As was lately proposed [42], differential co-expression network in which edges were weighted or dichotomized by direct differential co-expression measures (such as *dC *in the present study) have distinctive topological features compared to traditional co-expression networks, so co-expression network-based strategies could not directly transfer to differential co-expression network studies. The *ad hoc *clustering algorithm by Gu et al. [41] could possibly be adapted for exploration of differential co-expression network, and it is of interest to see continuous improvement of these algorithms and hopefully a comparative evaluation of these related approaches will come out soon.

## Conclusions

In this study, by integrating the protein interaction data and gene differential co-expression information, we sought to identify dynamic protein interaction modules from a hepatocellular carcinoma stage-wise expression dataset. We established the validity of searching for subnetworks from seeds of differential co-expressed gene pairs in contrast to traditional differential expression genes. Moreover, by examining the differential co-expression patterns associated with single stage-transitions or whole progression process, we revealed dynamic rewiring of protein interaction pairs and temporal activation/dismissal of pivotal functions in human hepatocellular carcinoma progression. Our framework has generated a valuable set of plausibly implicated genes and protein interaction pairs for follow-up human hepatocellular carcinoma investigations.

## Materials and methods

### Gene expression profiles and protein interaction network

The HCC gene expression dataset GSE6764 [25] was obtained from Gene Expression Omnibus (GEO) [43]. It contains 20,068 genes and 75 samples. Three samples from cirrhotic liver tissue of non-HCC patients were excluded; the remaining 72 samples were classified into five stages of HCC development: Normal (N), Cirrhosis (C), Dysplasia (D), Early HCC (E), and Advanced HCC (A). The numbers of samples included in these stages were 10, 10, 17, 18, and 17, respectively.

Protein interaction pairs were downloaded on September 14, 2012 from the Protein Interaction Network Analysis (PINA) [44], which collected 70,297 protein interaction pairs between 12,373 proteins. After matching them with dataset GSE6764, we had a protein interaction network of 64,865 pairs between 10,953 proteins.

Another independent HCC gene expression dataset (GSE14323) [45] was used to verify the N-C transition results produced from the primary dataset GSE6764. Nineteen normal and 41 cirrhotic tissue samples of this dataset were used.

### HCC-related gene sets

We compiled three sets of HCC-related genes as gold standards to evaluate the relevance of our results. The HCV-protein-binding (HCB) proteins were downloaded from the Hepatitis C Virus Protein Interaction Database [46] on October 17, 2012. The Cancer Gene Consensus (CGC) set included the cancer-mutated genes last updated on March 15, 2012 [47]. The HCC-responsive set (HCR) was a compendium of DEGs reported in HCC studies, compiled by querying the gene set database MSigDB [48] with the keywords "hepatocellular AND carcinoma." Mapping to our protein interaction network, we were left with 393 HCB, 385 CGC, and 4,791 HCR genes, respectively.

### Discovery of differential co-expression subnetworks in HCC

We calculated the Pearson correlation coefficient (*r*) for each gene pair under each HCC stage. Then, the Pearson correlation coefficients were transformed using Fisher's transformation [49]. Fisher's transformation could achieve a soft thresholding of the original *r *values so that the larger *r *values were emphasized while the smaller ones were downplayed. The transformed correlation value (*R_k _*) was calculated as in equation 1:

(1) $$R_{k}=0.5*\text{ln}\frac{\left(1+r_{k}\right)}{\left(1-r_{k}\right)}$$

where $r_{k}$ was untransformed Pearson correlation coefficient values with *k *being 1, 2, 3, 4, or 5 (corresponding to the five HCC stages). After the Fisher's transformation, the transformed correlation value of each subsequent stage was subtracted from the counterpart values in the preceding stage, and a differential correlation value (*dC*) was obtained as calculated by equation 2. Consequently, four *dC *values corresponding to the four stage transitions were assigned to each gene pair.

(2) $$dC\left(k\right)=R_{k+1}-R_{k}$$

As in the initial work [8] and our previous work [24], we searched for dense modules in protein interaction network from a beginning set of seeds. The procedure started from each of several initial network modules formed by top ranked DCPs (seeds) and ended with a combination of the iteratively expanded modules. At each iterative step, the module was growing outwardly by absorbing a directly-connecting edge with the maximum absolute *dC *value, and it was assessed for its module score - the average absolute *dC *value. Most often, the module score would decrease with the subnetwork expansion. The iteration continued if and only if the decreasing rate of the module score was not greater than *delta *and ended as the iteration exceeded 100 times. The strategy of the decreasing module score rate was applied to this approach, as we observed that the module scores often decreased in the first few steps during the expansion procedure. By applying the decreasing rate of module score, the chances of getting stuck at the early iteration stages were diminished; fragmented, small sized modules comprising most of the starting seeds could most likely be avoided. The upper-limit of iteration cycles was set at 100 to control the size of the resulting network modules. This subnetwork-searching algorithm was named the "edgewise dense module searching" (eDMS). An R script for eDMS is available upon request.

As stated in the previous work [8], *delta *is decisive in determining the final modules in network search. We followed the procedure from our previous work [24] to analyze a spectrum of *delta *(from 0 to 0.1 with an increase interval of 0.01). We then selected the optimal *delta *based on the overall module score - the average module score of disconnected components after eDMS ends. As in the previous work, we removed the disconnected components with less than five comprising genes before we reported the final subnetwork.

### Functional annotation for a set of genes

GO [27] term enrichment analyses (in the "Biological Process" aspect) were performed using the hyper-geometric test provided in the R package GOstats [50], with the genes in the global protein interaction network taken as the universal background. For each GO term, the numbers of annotated genes from the background gene set and the foreground set (e.g. from a subnetwork or a set of hubs) were each identified. Then, a *p*-value indicative of the enrichment level of the GO term in question was calculated. After removing GO terms with four or less genes annotated from the foreground set, we adjusted the remaining GO terms' *p*-values using the Benjamini-Hochberg method [51]. GO terms with adjusted *p*-value larger than 0.001 (Clusters I, II, III, and V) or 0.01 (Cluster IV) were removed and only the most specific terms (leaf terms) of the remaining term set were reported.

## List of abbreviations used

CGC: Cancer Gene Consensus

GO: Gene Ontology

DEG: differentially expressed gene

DCG: differentially co-expressed gene

DCP: differentially co-expressed gene pair

HCB: HCV-protein-binding

HCC: Hepatocellular carcinoma

HCR: HCC-responsive

HCV: *Hepatitis C *virus

## Competing interests

The authors declare that they have no competing interests.

## Authors' contributions

ZZ, HY, and YYL conceived of the study and collected the data. HY performed the computational coding and implementation. HY and CCL conducted data analysis. HY, CCL, and ZZ drafted the manuscript. All authors read and approved the final manuscript.

## Supplementary Material

Additional File 1 **Supplementary results**. This file contains all supplementary results that are not covered in the other additional files. Explanatory text, small tables, and small figures are included in this file. Click here for file

Additional file 2 **Seeds for subnetwork searches**. This file documents the seed DCPs used for the four HCC-transition subnetwork searches. Click here for file

Additional file 3 **Transition-wise differential co-expression protein subnetworks**. This file includes the visual display of all four transition-wise differential co-expression subnetworks. Click here for file

Additional file 4 **Edge statistics of transition-wise differential co-expression protein subnetworks**. This file includes the statistics associated with all edges of the transition-wise differential co-expression subnetworks. Click here for file

Additional file 5 **Process-wise clusters of dynamic protein interaction modules**. This file includes the visual display of the six clusters of process-wise differential co-expression protein interaction modules. Click here for file

Additional file 6 **Edge statistics of process-wise differential co-expression protein modules**. This file includes the expression correlation values (***r***) and differential co-expression values (***dC***) associated with edges from the six clusters of process-wise protein modules. Click here for file

## Declarations

The publication costs for this article were funded by the corresponding author.

This article has been published as part of *BMC Systems Biology *Volume 7 Supplement 5, 2013: Selected articles from the International Conference on Intelligent Biology and Medicine (ICIBM 2013): Systems Biology. The full contents of the supplement are available online at http://www.biomedcentral.com/bmcsystbiol/supplements/7/S5.
