# Supplementary material for: Dynamic protein interaction modules in human hepatocellular carcinoma progression
Source: BMC Syst Biol. 2013 Dec 9;7(Suppl 5):S2. doi: 10.1186/1752-0509-7-S5-S2 (PMC4029569; doi:10.1186/1752-0509-7-S5-S2)
Supplement: Additional File 1 — Supplementary results. This file contains all supplementary results that are not covered in the other additional files. Explanatory text, small tables, and small figures are included in this file. [file 1752-0509-7-S5-S2-S1.doc]

**Supplementary results to “Dynamic protein interaction modules in human hepatocellular carcinoma progression”**

[A portion of PPIs with distinctively larger *dC* values 1](#__RefHeading___Toc365404582)

[Enrichment of HCC-related genes in seed genes 2](#__RefHeading___Toc365404583)

[Enrichment of differential co-expression in transition-wise subnetworks 3](#__RefHeading___Toc365404584)

[Functional terms enriched within subnetwork hubs 5](#__RefHeading___Toc365404585)

[Comparisons of transition-wise protein subnetworks 7](#__RefHeading___Toc365404586)

[Proteins interfacing with alternative interaction partner groups 8](#__RefHeading___Toc365404587)

[Putative precedence of differential co-expression over differential expression 11](#__RefHeading___Toc365404588)

# A portion of PPIs with distinctively larger *dC* values

We derived the differential correlation value (*dC*) for each the PPI at each HCC stage transition, so, in this way, each PPI pair was described with a vector of four *dC* values. We assumed a small portion of PPIs should have unusually larger *dC* values than the majority PPIs. To prove this assumption, we resorted to the multivariate outlier analysis algorithm MCD (minimum covariance determinant) to test a null assumption – is the *dC* data matrix made up with a single multivariate normal distribution? This null hypothesis means that all PPIs were in principal similar in terms of differential co-expression, and the corresponding alternative hypothesis means that a portion of PPIs were significantly different from the rest majority by having exceptionally larger *dC* values. The chisq.plot function from the R package ‘mvoutlier’ (<http://cran.r-project.org/web/packages/mvoutlier/index.html>) was adopted to draw a quantile-quantile plot of the data points’ distances to the estimated distribution center (Figure 1). It is clearly shown that quite many data points deviated from the theoretical Chi-squared distribution, implying that some PPI pairs did have distinctively larger *dC* values than the majority PPI pairs. This implication substantiated our subsequent searches of protein interaction subnetworks that were seeded from the highly differentially co-expressed PPIs.


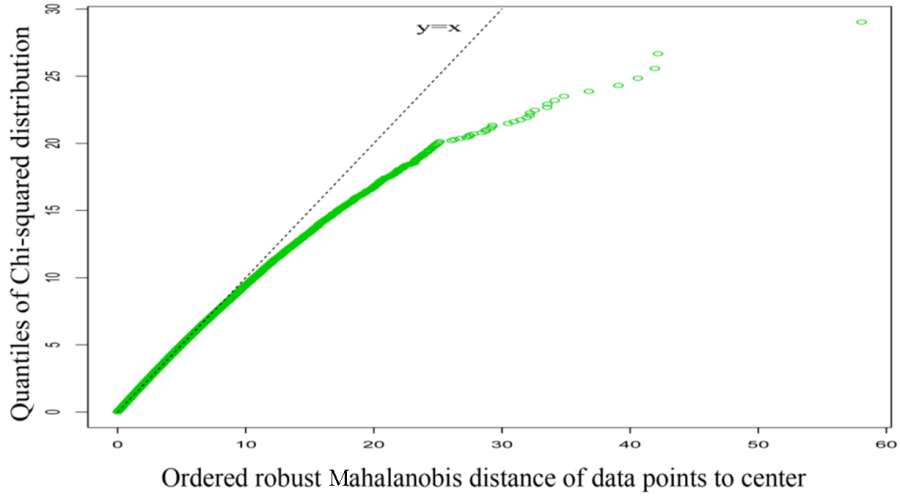


**Figure 1.Multivariate outlier analysis of differential correlation data matrix.** If the *dC* vectors of all PPIs follow a same multivariate normal distribution, the ordered robust Mahalanobis distances (abscissa) should be distributed similarly as the quantiles of chi-squared distribution (ordinate). The apparent downward deviation from the diagonal line implies a sizeable portion of PPI pairs have distinctively larger *dC* values.

# Enrichment of HCC-related genes in seed genes

The top ranked seeds of differentially expressed genes (DEGs) or differentially co-expressed genes (DCGs) derived from differentially co-expressed pairs were compared in terms of their coverage of HCC-related genes: HCV-binding genes/proteins (shown in manuscript), cancer-mutatedgenes (Table 1), or HCC-responsive genes (Table 2). It was found that in general top-DCGs were superior to DEGs in enriching these HCC-related genes.

**Table 1.Proportion of cancer-mutated genes in top ranked genes.**

| **Top-level ^a^** | **Gene Set** | **HCC transitions ^b^** | | | |
| --- | --- | --- | --- | --- | --- |
|  |  | **N-CI** | **CI-DY** | **DY-E** | **E-A** |
| 0.1% | DEG | 1/11 | 0/11 | 0/11 | 0/11 |
|  | DCG | 4/63 | 5/65 | 9/60^***^ | 6/63^*^ |
| 0.5% | DEG | 1/55 | 1/55 | 1/55 | 4/55 |
|  | DCG | 26/290^***^ | 25/279^***^ | 26/287^***^ | 21/288^*^ |
| 1.0% | DEG | 3/110 | 4/110 | 7/110 | 9/110^*^ |
|  | DCG | 43/524^***^ | 47/538^***^ | 41/523^***^ | 46/559^***^ |

In each cell, the proportion is shown as ″x/y″, with y for top ranked genes and x for HCC-related genes. Enrichment *p*-value < 0.01 (^*^) or < 0.0001 (^***^) are marked.

^a^top-level is the fraction of the top-ranked genes/pairs in the total network nodes/edges. For differential expression genes (DEG), we used the node-wise fraction; for differential co-expression genes (DCGs) deriving from pairs, we used the edge-wise fraction. A same fraction of pairs involve a greater number of genes.

^b^Transition names: Normal to Cirrhosis (N-C), Cirrhosis to Dysplasia (C-D), Dysplasia to Early HCC (D-E), and Early HCC to Advanced HCC (E-A).

**Table 2.Proportion of HCC-responsive genes in top ranked genes.**

| **Top-level^a^** | **Gene Set^b^** | **HCC transitions** | | | |
| --- | --- | --- | --- | --- | --- |
|  |  | **N-CI** | **CI-DY** | **DY-E** | **E-A** |
| 0.1% | DEG | 5/11 | 3/11 | 7/11 | 4/11 |
|  | DCG | 26/63 | 34/65 | 29/60 | 32/63 |
| 0.5% | DEG | 21/55 | 26/55 | 29/55 | 26/55 |
|  | DCG | 157/290^***^ | 154/279^***^ | 165/287^***^ | 154/288^*^ |
| 1.0% | DEG | 50/110 | 58/110 | 57/110 | 50/110 |
|  | DCG | 284/524^***^ | 296/538^***^ | 301/523^***^ | 310/559^***^ |

All symbols in Table 2 have the same meaning as in Table 1.

# Enrichment of differential co-expression in transition-wise subnetworks

The properties of four transition-wise subnetworks are summarized in Table 3. In each subnetwork, one edge is associated with a *dC* value derived from a pair of Pearson correlation coefficients (PCC). The maximum absolute PCC of each pair was deemed as the correlation, and the minimum of the correlations over all edges in one subnetwork was shown in the column the “Min *r*” column of Table 3. In another aspect, we sorted the edge-wise absolute *dC* values of the global PIN decreasingly (the larger *dC*, the smallest rank) and identified the percentile of the maximum *dC* in a subnetwork (column “Max *dC*’s percentile” in Table 3). The bias of subnetwork *dCs’* percentiles (ranks) towards the small values can be seen in Figure 2. Taken together, these results indicated that the PPI relations in our extracted subnetworks are remarkable in stage-wise co-expression and transition-wise differential co-expression as well.

**Table 3.Four transition-wise differential co-expression PIN subnetwork**s.

| **Transition ^a^** | **# nodes** | **# edges** | **HCC-related genes ^b^** | | | **Min *r*** | **Max *dC* percentile** | **Hubs** ^c^ |
| --- | --- | --- | --- | --- | --- | --- | --- | --- |
|  |  |  | **CGC** | **HCR** | **HCB** |  |  |  |
| N-C | 307 | 310 | 29**^***^** | 154**^*^** | 28**^***^** | 0.66 | 1.6% | *GRB2,YWHAZ, CSNK2A1, SRRM2,* ***PIK3R1****, HCK,* ***EP300****, YWHAG, CSNK2A2, MCC,* ***APC****,* ***CDKN2A****, ARRB1, SUMO2,ESR1, PPP1CC, TSC22D1* |
| C-D | 102 | 103 | 10**^*^** | 44 | 13**^***^** | 0.65 | 1.0% | *APC, GFI1B, NEDD4, UBE2D3* |
| D-E | 104 | 103 | 13**^***^** | 58**^*^** | 10**^*^** | 0.54 | 0.96% | *YWHAZ, PIK3R1, IKBKE* |
| E-A | 103 | 102 | 11**^*^** | 60**^*^** | 9**^*^** | 0.50 | 1.5% | *FYN ,* ***EGFR****, YWHAG, IKBKG, PRMT1* |

^a^Transition names: Normal to Cirrhosis (N-C), Cirrhosis to Dysplasia (C-D), Dysplasia to Early HCC (D-E), and Early HCC to Advanced HCC (E-A).

^b^see main text for explanation of the three related gene sets. Significance levels of HCC-related gene enrichment are labeled by ^*^(*p*< 0.01) and ^***^(*p*< 0.0001).

^c^hubs are genes with six or more connected neighbors. HCV-binding genes (underlined) and cancer-mutated genes (**bold**) are marked.

**Figure 2. Distribution of *dC* Ranks of subnetwork edges.**

# Functional terms enriched within subnetwork hubs

Following the example in our previous work , we defined proteins with six or more connections as hub proteins, and thus obtained a total of 25 hubs in the four transition-wise subnetworks. Using the function enrichment analysis method described in Materials and methods of the manuscript, we retrieved Gene Ontology (Biological Process) functional terms enriched in these hub proteins (Table 4).

**Table 4. Gene Ontology (Biological Process) functional terms enriched within hubs of HCC transition-wise** subnetworks.

| **GOBPID** | **Term** | **Odds Ratio** | **# expec-ted genes** | **# genes** | **Adjusted *p*-value** |
| --- | --- | --- | --- | --- | --- |
| GO:0007166 | cell surface receptor signaling pathway | 6.6 | 4.7 | 15 | 0.00012 |
| GO:0046822 | regulation of nucleocytoplasmic transport | 20.0 | 0.3 | 5 | 0.00016 |
| GO:0006974 | response to DNA damage stimulus | 8.8 | 1.3 | 8 | 0.00019 |
| GO:0044419 | interspecies interaction between organisms | 10.3 | 0.9 | 7 | 0.00019 |
| GO:0051239 | regulation of multicellular organismal process | 5.8 | 3.5 | 12 | 0.00024 |
| GO:0009967 | positive regulation of signal transduction | 7.9 | 1.4 | 8 | 0.00026 |
| GO:0010638 | positive regulation of organelle organization | 15.0 | 0.4 | 5 | 0.00026 |
| GO:0051090 | regulation of sequence-specific DNA binding transcription factor activity | 11.2 | 0.7 | 6 | 0.00026 |
| GO:0040011 | locomotion | 5.9 | 2.6 | 10 | 0.00040 |
| GO:0007409 | axonogenesis | 8.2 | 1.1 | 7 | 0.00040 |
| GO:0006468 | protein phosphorylation | 5.9 | 2.6 | 10 | 0.00041 |
| GO:0009968 | negative regulation of signal transduction | 7.9 | 1.2 | 7 | 0.00050 |
| GO:0010627 | regulation of intracellular protein kinase cascade | 7.8 | 1.2 | 7 | 0.00054 |
| GO:0051247 | positive regulation of protein metabolic process | 7.7 | 1.2 | 7 | 0.00054 |
| GO:0002757 | immune response-activating signal transduction | 11.9 | 0.5 | 5 | 0.00057 |
| GO:0000278 | mitotic cell cycle | 6.6 | 1.7 | 8 | 0.00058 |
| GO:0030168 | platelet activation | 11.7 | 0.5 | 5 | 0.00058 |
| GO:0042981 | regulation of apoptotic process | 5.5 | 2.7 | 10 | 0.00058 |

# Comparisons of transition-wise protein subnetworks

One differential co-expression protein interaction subnetwork was extracted for each HCC stage transition, and the overlap of nodes/edges was examined for each pair of transitions (Table 5). Altogether we observed 16 differential co-expression PPIs recurrent in multiple subnetworks, with two involving the N-C and the E-A transitions (Table 6), and the other 14 involving the N-C and C-D transitions (Table 7).

**Table 5. Overlap of nodes or edges between transition-wise subnetworks.** Node comparison: lower-left triangle; edge comparison: upper-right triangle.

|  | **N-C** | **C-D** | **D-E** | **E-A** |
| --- | --- | --- | --- | --- |
| N-C | - | 14 (13.6%) | 0 | 2 (2.0%) |
| C-D | 29 (28.4%) | - | 0 | 0 |
| D-E | 12 (11.5%) | 1 (1.0%) | - | 0 |
| E-A | 10 (9.7%) | 5 (4.9%) | 1 (0.9%) | - |

Transition names: Normal to Cirrhosis (N-C), Cirrhosis to Dysplasia (C-D), Dysplasia to Early HCC (D-E), and Early HCC to Advanced HCC (E-A).

**Table 6.Differential co-expression protein interaction pairs shared between the first and the last HCC stage-transitions**.

| **Gene 1** | **Gene 2** | **Expression correlation** ^a^ | | | | **Differential correlation** ^b^ | | |
| --- | --- | --- | --- | --- | --- | --- | --- | --- |
|  |  | **N** | **C** | **E** | **A** | | **N-C** | **E-A** |
| *BRAF* | *YWHAG* | 0.954 | -0.007 | 0.914 | 0.131 | | -1.88 | -1.42 |
| *CRTC2* | *YWHAG* | 0.852 ^c^ | -0.304 | 0.802 ^d^ | 0.102 | | -1.58 | -1.00 |

^a^ Expression correlation is associated with HCC stages (N for Normal, C for Cirrhosis, E for Early HCC, and A for Advanced HCC).

^b^ Differential correlation is associated with HCC stage transitions: Normal to Cirrhosis (N-C) and Early HCC to Advanced HCC (E-A).

^c^ Expression correlation value of 0.852 in the normal stage corresponds to a *p*-value of 0.001 7 and a Benjamin-Horchberg adjusted *q*-value of 0.06.

^d^ Expression correlation value of 0.802 in the normal stage corresponds to a *p*-value <1E-4 and a Benjamin-Horchberg adjusted *p*-value of 0.025.

**Table 7.Differential co-expression protein interaction pairs shared among the first two** HCC stage-transitions.

| **Gene 1** | **Gene 2** | **Expression correlation** ^a^ | | | **Differential correlation** ^b^ | |
| --- | --- | --- | --- | --- | --- | --- |
|  |  | **N** | **C** | **D** | **N-C** | **C-D** |
| *CTNNA1* | *APC* | 0.594 | -0.894 | 0.453 | -2.12 | 1.93 |
| *NUPL1* | *APC* | 0.410 | -0.897 | -0.018 | -1.89 | 1.43 |
| *ZFP106* | *APC* | -0.004 | 0.946 | 0.369 | 1.79 | -1.40 |
| *CYTH2* | *APC* | -0.222 | 0.945 | 0.232 | 2.00 | -1.54 |
| *HNRNPF* | *APC* | -0.021 | 0.953 | -0.144 | 1.88 | -2.00 |
| *TOB1* | *HNRNPF* | -0.048 | 0.930 | 0.084 | 1.70 | -1.57 |
| *CYTH2* | *ARRB2* | 0.512 | 0.977 | 0.578 | 1.65 | -1.56 |
| *ARRB1* | *CXCR2* | 0.263 | -0.917 | -0.168 | -1.83 | 1.39 |
| *NSF* | *ARRB1* | -0.128 | 0.931 | -0.078 | 1.79 | -1.74 |
| *BCR* | *CRKL* | 0.143 | 0.958 | 0.312 | 1.77 | -1.59 |
| *CXCR2* | *CXCR1* | 0.309 | 0.983 | 0.657 | 2.04 | -1.57 |
| *PDCL* | *IKBKG* | -0.498 | 0.772 ^c^ | -0.439 | 1.57 | -1.49 |
| *NSF* | *SNAP29* | 0.005 | -0.923 | 0.347 | -1.61 | 1.96 |
| *TPR* | *NUP153* | 0.545 | -0.826 | 0.284 | -1.78 | 1.46 |

^a^ Expression correlation is associated with HCC stages (N for Normal, C for Cirrhosis, and D for Dysplasia).

^b^ Differential correlation is associated with HCC stage transitions: Normal to Cirrhosis (N-C) and Cirrhosis to Dysplasia (C-D).

^a^ Expression correlation is associated with HCC stages (N for Normal, C for Cirrhosis, E for Early HCC, and A for Advanced HCC).

^c^ The minimal correlation value of these fourteen PPI pairs in the Cirrhosis stage corresponds to a *p*-value of 0.009 and an adjusted *p*-value of 0.23 as corrected by the Benjamin-Hochberg method.

# Proteins interfacing with alternative interaction partner groups

Twenty-seven interfacing proteins were found interacting with partners from different dynamic co-expression clusters. The topological features of these interfacing proteins in the process-wise subnetwork are shown in Table 8. Of these 27 proteins, five bind with Hepatitis C virus proteins, and 17 are covered in MSigDB HCC-responsive datasets.

**Table 8.Interfacing proteins that interact with partners from different dynamic co-expression clusters**.

| **protein** | **cluster** | **Cluster**  **Degree** | | **Degree** | | ***Degree Rank*** | | ***Between-nessRank*** | | **HCV-binding protein** | | | **MSigDB gene set: "Hepatocellular AND Carcinoma"** |
| --- | --- | --- | --- | --- | --- | --- | --- | --- | --- | --- | --- | --- | --- |
| **YWHAZ** | cluster 1 | | **4** | | **33** | | *1* | | 2 | | CORE | YAMASHITA_LIVER_CANCER_STEM_CELL_UP; PATIL_LIVER_CANCER | |
|  | cluster 2 | | **4** | |  |  |  |  |  |  |  |  |  |
|  | cluster 3 | | **4** | |  |  |  |  |  |  |  |  |  |
|  | cluster 4 | | **5** | |  |  |  |  |  |  |  |  |  |
|  | cluster 5 | | **7** | |  |  |  |  |  |  |  |  |  |
|  | cluster 6 | | **9** | |  |  |  |  |  |  |  |  |  |
| TSC22D1 | cluster 1 | | **6** | | **17** | | *2* | | 62 | |  |  | |
|  | cluster 3 | | **11** | |  |  |  |  |  |  |  |  |  |
| *APC* | cluster 1 | | **8** | | **16** | | *5* | | 1 | |  |  | |
|  | cluster 3 | | **7** | |  |  |  |  |  |  |  |  |  |
|  | cluster 4 | | **1** | |  |  |  |  |  |  |  |  |  |
| YWHAG | cluster 1 | | **7** | | **16** | | *5* | | 9 | |  | ACEVEDO_LIVER_CANCER_UP | |
|  | cluster 4 | | **4** | |  |  |  |  |  |  |  |  |  |
|  | cluster 6 | | **5** | |  |  |  |  |  |  |  |  |  |
| IKBKG | cluster 2 | | **1** | | **16** | | *5* | | 3 | |  |  | |
|  | cluster 3 | | **2** | |  |  |  |  |  |  |  |  |  |
|  | cluster 4 | | **8** | |  |  |  |  |  |  |  |  |  |
|  | cluster 5 | | **5** | |  |  |  |  |  |  |  |  |  |
| ARRB1 | cluster 1 | | **9** | | **14** | | *6* | | 6 | |  |  | |
|  | cluster 3 | | **5** | |  |  |  |  |  |  |  |  |  |
| ESR1 | cluster 1 | | **5** | | **11** | | *7* | | 28 | |  | SAKAI_CHRONIC_HEPATITIS_VS_LIVER_CANCER_DN; YAMASHITA_LIVER_CANCER_STEM_CELL_UP; YAMASHITA_LIVER_CANCER  _STEM_CELL_DN; YAMASHITA_LIVER_CANCER_STEM_CELL_DN | |
|  | cluster 3 | | **6** | |  |  |  |  |  |  |  |  |  |
| **FYN** | cluster 1 | | **1** | | **10** | | *9* | | 33 | | NS5A | BOYAULT_LIVER_CANCER_SUBCLASS_G123_DN; ACEVEDO_METHYLATED_IN_LIVER_CANCER_DN | |
|  | cluster 4 | | **9** | |  |  |  |  |  |  |  |  |  |
| **GRB2** | cluster 1 | | **5** | | **10** | | *9* | | 7 | | NS5A |  | |
|  | cluster 3 | | **5** | |  |  |  |  |  |  |  |  |  |
| NEDD4 | cluster 2 | | **6** | | **9** | | *10* | | 16 | |  | BOYAULT_LIVER_CANCER_SUBCLASS_G56_UP | |
|  | cluster 3 | | **1** | |  |  |  |  |  |  |  |  |  |
|  | cluster 4 | | **2** | |  |  |  |  |  |  |  |  |  |
| ***EP300*** | cluster 2 | | **3** | | **8** | | *11* | | 11 | | CORE | HOSHIDA_LIVER_CANCER_SUBCLASS_S2 | |
|  | cluster 3 | | **5** | |  |  |  |  |  |  |  |  |  |
| *CDKN2A* | cluster 1 | | **4** | | **6** | | *17* | | 88 | |  | YAMASHITA_LIVER_CANCER_STEM_CELL_UP; YAMASHITA_LIVER_CANCER_STEM_CELL_DN; ACEVEDO_LIVER_CANCER_WITH_H3K27ME3_DN | |
|  | cluster 3 | | **2** | |  |  |  |  |  |  |  |  |  |
| HGS | cluster 2 | | **5** | | **6** | | *17* | | 35 | |  | BOYAULT_LIVER_CANCER_SUBCLASS_G3_UP; PATIL_LIVER_CANCER | |
|  | cluster 3 | | **1** | |  |  |  |  |  |  |  |  |  |
| NUP153 | cluster 1 | | **1** | | **5** | | *22* | | 4 | |  | HOSHIDA_LIVER_CANCER_SUBCLASS_S2; ACEVEDO_LIVER_CANCER_UP | |
|  | cluster 3 | | **2** | |  |  |  |  |  |  |  |  |  |
|  | cluster 4 | | **2** | |  |  |  |  |  |  |  |  |  |
| ARRB2 | cluster 1 | | **1** | | **4** | | *33* | | 15 | |  |  | |
|  | cluster 2 | | **2** | |  |  |  |  |  |  |  |  |  |
|  | cluster 3 | | **1** | |  |  |  |  |  |  |  |  |  |
| MEPCE | cluster 1 | | **1** | | **4** | | *33* | | 22 | |  |  | |
|  | cluster 3 | | **3** | |  |  |  |  |  |  |  |  |  |
| EEF1D | cluster 3 | | **2** | | **4** | | *33* | | 29 | |  |  | |
|  | cluster 4 | | **2** | |  |  |  |  |  |  |  |  |  |
| *BRCA1* | cluster 2 | | **1** | | **4** | | *33* | | 18 | |  | RADAEVA_RESPONSE_TO_IFNA1_UP; LEE_LIVER_CANCER_CIPROFIBRATE_UP | |
|  | cluster 3 | | **1** | |  |  |  |  |  |  |  |  |  |
|  | cluster 4 | | **2** | |  |  |  |  |  |  |  |  |  |
| SMAD2 | cluster 3 | | **3** | | **4** | | *33* | | 5 | |  | BREUHAHN_GROWTH_FACTOR_SIGNALING_IN_LIVER_CANCER; BOYAULT_LIVER_CANCER_SUBCLASS_G3_UP; HOSHIDA_LIVER_CANCER_SUBCLASS_S1 | |
|  | cluster 4 | | **1** | |  |  |  |  |  |  |  |  |  |
| *TPR* | cluster 1 | | **2** | | **3** | | *56* | | 19 | |  | LEE_LIVER_CANCER_MYC_UP; LEE_LIVER_CANCER_MYC_E2F1_UP; BOYAULT_LIVER_CANCER_SUBCLASS_G1_UP; HOSHIDA_LIVER_CANCER_ SUBCLASS_S2; PATIL_LIVER_CANCER | |
|  | cluster 3 | | **1** | |  |  |  |  |  |  |  |  |  |
| CYTH2 | cluster 1 | | **1** | | **3** | | *56* | | 14 | |  |  | |
|  | cluster 3 | | **2** | |  |  |  |  |  |  |  |  |  |
| **SMURF2** | cluster 2 | | **2** | | **3** | | *56* | | 45 | | NS3 | WANG_RECURRENT_LIVER_CANCER_UP; COULOUARN_TEMPORAL_TGFB1_SIGNATURE_UP | |
|  | cluster 3 | | **1** | |  |  |  |  |  |  |  |  |  |
| TSPYL2 | cluster 2 | | **2** | | **3** | | *56* | | 50 | |  | COULOUARN_TEMPORAL_TGFB1_SIGNATURE_DN | |
|  | cluster 3 | | **1** | |  |  |  |  |  |  |  |  |  |
| TUBA1A | cluster 4 | | **2** | | **3** | | *56* | | 24 | |  | PATIL_LIVER_CANCER;ACEVEDO_LIVER_CANCER_UP | |
|  | cluster 6 | | **1** | |  |  |  |  |  |  |  |  |  |
| CRKL | cluster 1 | | **2** | | **3** | | *56* | | 46 | |  |  | |
|  | cluster 3 | | **1** | |  |  |  |  |  |  |  |  |  |
| ADRB2 | cluster 1 | | **1** | | **2** | | *110* | | 109 | |  | CHIANG_LIVER_CANCER_SUBCLASS_CTNNB1_UP; CHIANG_LIVER_CANCER_SUBCLASS_PROLIFERATION_DN | |
|  | cluster 2 | | **1** | |  |  |  |  |  |  |  |  | |
| ZFP106 | cluster 3 | | **1** | | **2** | | *110* | | 34 | |  | ACEVEDO_LIVER_CANCER_UP | |
|  | cluster 4 | | **1** | |  |  |  |  |  |  |  |  |  |

Underline: genes included in MSigDB’s HCC-responsive gene sets; Bold: HCV-binding proteins; *Italic*: cancer-mutated Genes from Cancer Gene Consensus.

# Putative precedence of differential co-expression over differential expression

We investigated genes which were conspicuous in both differential co-expression (DC) and differential expression (DE). We compared our DC results with the DE results from the original study , and found three genes, ESR1, LCK, and EEF1D, stood out from both the original study and the current study. Interestingly, these three genes all underwent an earlier differential co-expression then a later differential expression (Table 9). On the other hand, we compared our DC-subnetworks with the DE-subnetworks resolved from the same GSE6764 dataset , and found 21 common genes. Seventeen of these 21 common gene were differential co-expression at an earlier transition and then differential expression at a later transition; in particular, of the 14 genes differentially co-expressed at the N-C transition, two were simultaneously differential expression, while the other 12 were differential expression at later transitions (Table 10).

**Table 9.Genes shown as DE in previous study (Wurmbach et al. 2007) and DC in the current study.**

| **Gene** | **Transition ^a^** | | | |
| --- | --- | --- | --- | --- |
|  | N-C | C-D | D-E | E-A |
| **ESR1** | DC |  | DC | DE |
| **LCK** | DC | DE |  |  |
| **EEF1D** |  | DC | DE | DC |

^a^Transition names: Normal to Cirrhosis (N-C), Cirrhosis to Dysplasia (C-D), Dysplasia to Early HCC (D-E), and Early HCC to Advanced HCC (E-A).

**Table 10.Genes shown as DE in previous study (Zheng et al. 2011) and DC in the current study**.

| Gene | Transition ^a^ | | | |
| --- | --- | --- | --- | --- |
|  | N-C | C-D | D-E | E-A |
| BIRC5 | DC |  | DE | DE |
| CDC25C |  |  | DC | DE |
| CDC6 | DC |  |  | DE |
| CHEK1 | DC |  |  | DE |
| CKS2 |  |  |  | DE, DC |
| COL1A1 | DE | DE |  | DC |
| ESR1 | DC |  | DC | DE |
| FCGR2C | DC |  | DE |  |
| FGFR2 | DC |  | DE |  |
| HDAC4 | DC |  | DE |  |
| HLA-B | DE |  |  | DC |
| HMGA2 | DC |  |  | DE |
| LCK | DE, DC | DE |  |  |
| LGALS3BP | DE, DC |  |  |  |
| MCM3 | DC |  |  | DE |
| MT2A | DC |  | DE |  |
| PDGFRA | DE, DC | DE |  |  |
| PLK1 | DC |  |  | DE |
| PTPRC |  | DE |  | DC |
| PTTG1 |  | DC | DE | DE |
| STAT1 | DE |  | DC |  |

^a^ Transition names: Normal to Cirrhosis (N-C), Cirrhosis to Dysplasia (C-D), Dysplasia to Early HCC (D-E), and Early HCC to Advanced HCC (E-A).

1. Garrett RG: **The chi-square plot: a tools for multivariate outlier recognition**. *Journal of Geochemical Exploration* 1989, **32**(1/3):319-341.

2. Zheng S, Tansey WP, Hiebert SW, Zhao Z: **Integrative network analysis identifies key genes and pathways in the progression of hepatitis C virus induced hepatocellular carcinoma**. *BMC medical genomics* 2011, **4**:62.

3. Wurmbach E, Chen YB, Khitrov G, Zhang W, Roayaie S, Schwartz M, Fiel I, Thung S, Mazzaferro V, Bruix J *et al*: **Genome-wide molecular profiles of HCV-induced dysplasia and hepatocellular carcinoma**. *Hepatology* 2007, **45**(4):938-947.
