# Supplementary material for: Dynamic protein interaction modules in human hepatocellular carcinoma progression
Source: BMC Syst Biol. 2013 Dec 9;7(Suppl 5):S2. doi: 10.1186/1752-0509-7-S5-S2 (PMC4029569; doi:10.1186/1752-0509-7-S5-S2)
Supplement: Additional file 3 — Transition-wise differential co-expression protein subnetworks. This file includes the visual display of all four transition-wise differential co-expression subnetworks. [file 1752-0509-7-S5-S2-S3.PDF]

Supplementary Figure 1. Transition-wise differential co-expression protein subnetworks. Node color differentiates different node types: green for HCV-protein-binding proteins, red for hub nodes (degree $\geq$ 5), and yellow for both HCV-binding protein and hub. Edge color differentiates different edge types: red for correlation increase, blue for correlation decrease. **A**, the N-C (Normal-Cirrhosis) subnetwork with 307 nodes and 310 edges; **B**, the C-D (Cirrhosis-dysplasia) subnetwork with 102 nodes and 103 edges; **C**, the D-E (Cirrhosis-Early HCC) subnetwork with 104 nodes and 103 edges; and **D**, the E-A (Early HCC-Advanced HCC) subnetwork with 103 nodes and 102 edges.

A

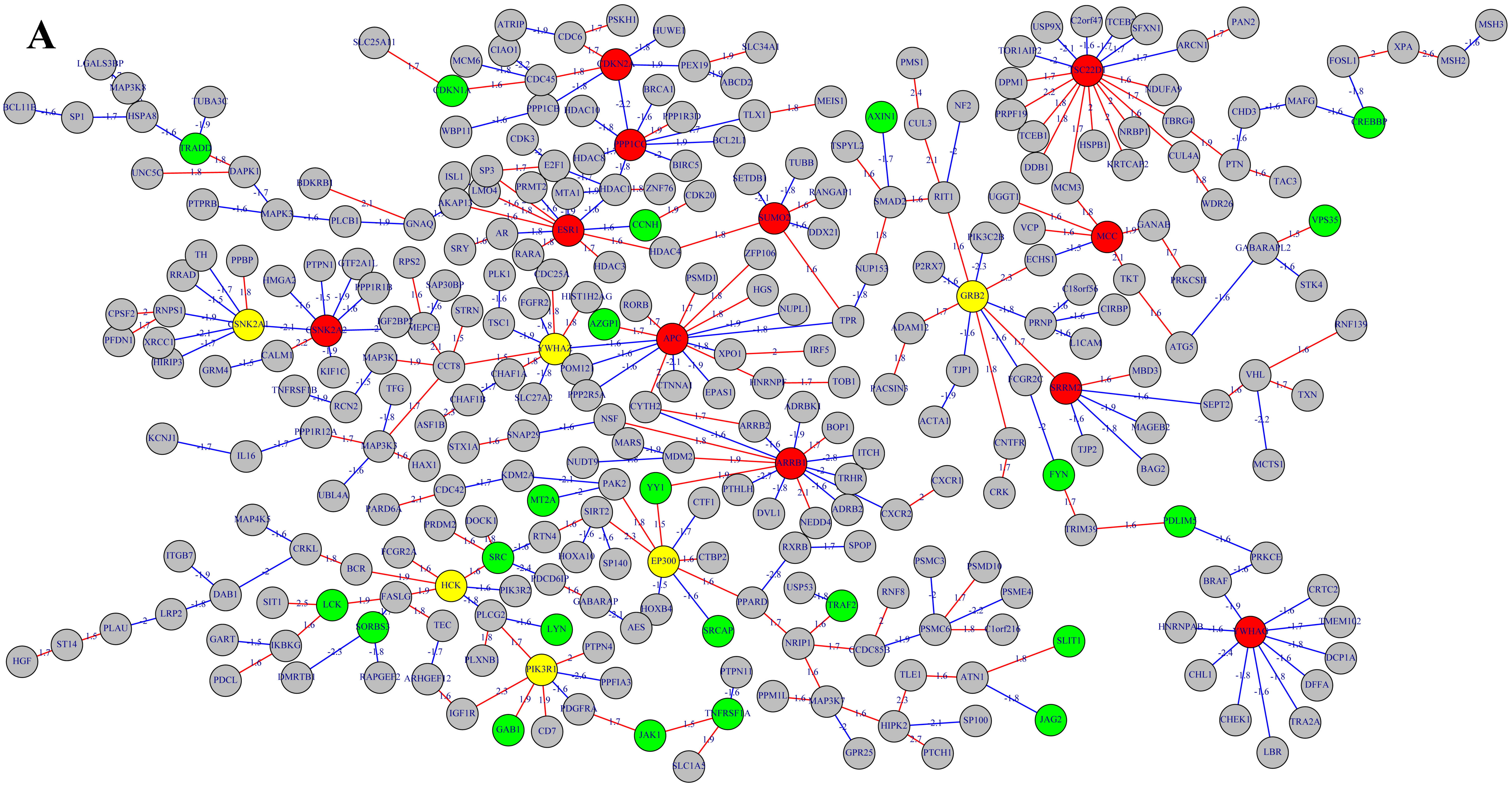

SLC25A11
CDKN1A
MCM6
CIAO1
ATRIP
CDC6
PSKH1
HUWE1
CDKN2A
PEX19
SLC34A1
ABCD2
CDC45
PPP1CB
WBP11
HDAC10
BRCA1
PPP1R3D
TLX1
MEIS1
PPP1CC
BCL2L1
BIRC5
CDK3
E2F1
HDAC8
SP3
ISL1
LMO4
AKAP13
PRMT2
MTA1
HDAC1
ZNF76
CDK20
ESR1
CCNH
AR
SRY
RARA
HDAC3
HDAC4
SUMO2
SETDB1
TUBB
RANGAP1
DDX21
TPR
LGALS3BP
MAP3K8
BCL11B
SP1
HSPA8
TUBA3C
TRADD
UNC5C
DAPK1
PTPRB
MAPK3
PLCB1
GNAQ
BDKRB1
TH
PPBP
RRAD
CPSF2
RNPS1
PFDN1
XRCC1
HIRIP3
CSNK2A1
CSNK2A2
HMGA2
PTPN1
GTF2A1L
PPP1R1B
GRM4
CALM1
KIF1C
RPS2
SAP30BP
MEPCE
IGF2BP1
STRN
CCT8
MAP3K1
RCN2
TNFRSF1B
TFG
PLK1
TSC1
FGFR2
CDC25A
HIST1H2AG
YWHAZ
AZGP1
RORB
APC
PSMD1
ZFP106
HGS
NUPL1
XPO1
IRF5
HNRNPF
TOB1
EPAS1
CTNNA1
POM121
PPP2R5A
CHAF1A
CHAF1B
SLC27A2
ASF1B
KCNJ1
IL16
PPP1R12A
MAP3K5
HAX1
UBL4A
STX1A
SNAP29
NSF
CYTH2
ARRB2
ADRBK1
BOP1
ITCH
ARRB1
MARS
MDM2
NUDT9
TRHR
PTHLH
DVL1
NEDD4
ADRB2
CXCR2
CXCR1
KDM2A
CDC42
PARD6A
PAK2
MT2A
YY1
CTF1
SIRT2
RTN4
HOXA10
SP140
EP300
CTBP2
SPOP
RXRB
USP53
TRAF2
PPARD
NRIP1
CCDC85B
RNF8
PSMC3
PSMD10
PSME4
PSMC6
C1orf216
MAP4K5
CRKL
ITGB7
DAB1
BCR
FCGR2A
HCK
PRDM2
DOCK1
SRC
PDCD6IP
GABARAP
PIK3R2
AES
HOXB4
SRCAP
LRP2
PLAU
ST14
HGF
SIT1
LCK
FASLG
SORBS3
TEC
GART
IKBKG
PDCL
DMRTB1
RAPGEF2
ARHGEF12
PLCG2
LYN
PLXNB
PIK3R1
PTPN4
PPFIA3
IGF1R
GAB1
CD7
PDGFRA
JAK1
TNFRSF1A
PTPN11
SLC1A5
PPM1L
MAP3K7
GPR25
HIPK2
TLE1
ATN1
SLIT1
JAG2
SP100
PTCH1
PMS1
CUL3
NF2
AXIN1
TSPYL2
SMAD2
RIT1
NUP153
P2RX7
PIK3C2B
GRB2
ADAM12
PACSIN3
TJP1
ACTA1
CNTFR
CRK
FCGR2C
FYN
TRIM39
PDLIM5
SRRM2
MBD3
TJP2
MAGEB2
BAG2
SEPT2
PRNP
C18orf56
CIRBP
L1CAM
ECHS1
UGGT1
VCP
MCC
GANAB
MCM3
PRKCSH
TKT
ATG5
USP9X
C2orf47
TCEB2
SFXN1
TOR1AIP2
TSC22D1
ARCN1
PAN2
DPM1
PRPF19
TCEB1
DDB1
HSPB1
NRBP1
KRTCAP2
NDUFA9
TBRG4
CUL4A
WDR26
PTN
TAC3
CHD3
MAFG
CREBBP
FOSL1
XPA
MSH2
MSH3
VPS35
GABARAPL2
STK4
RNF139
VHL
TXN
MCTS1
PRKCE
BRAF
CRTC2
HNRNPAB
YWHAG
TMEM102
DCP1A
DFFA
TRA2A
LBR
CHEK1
CHL1

B

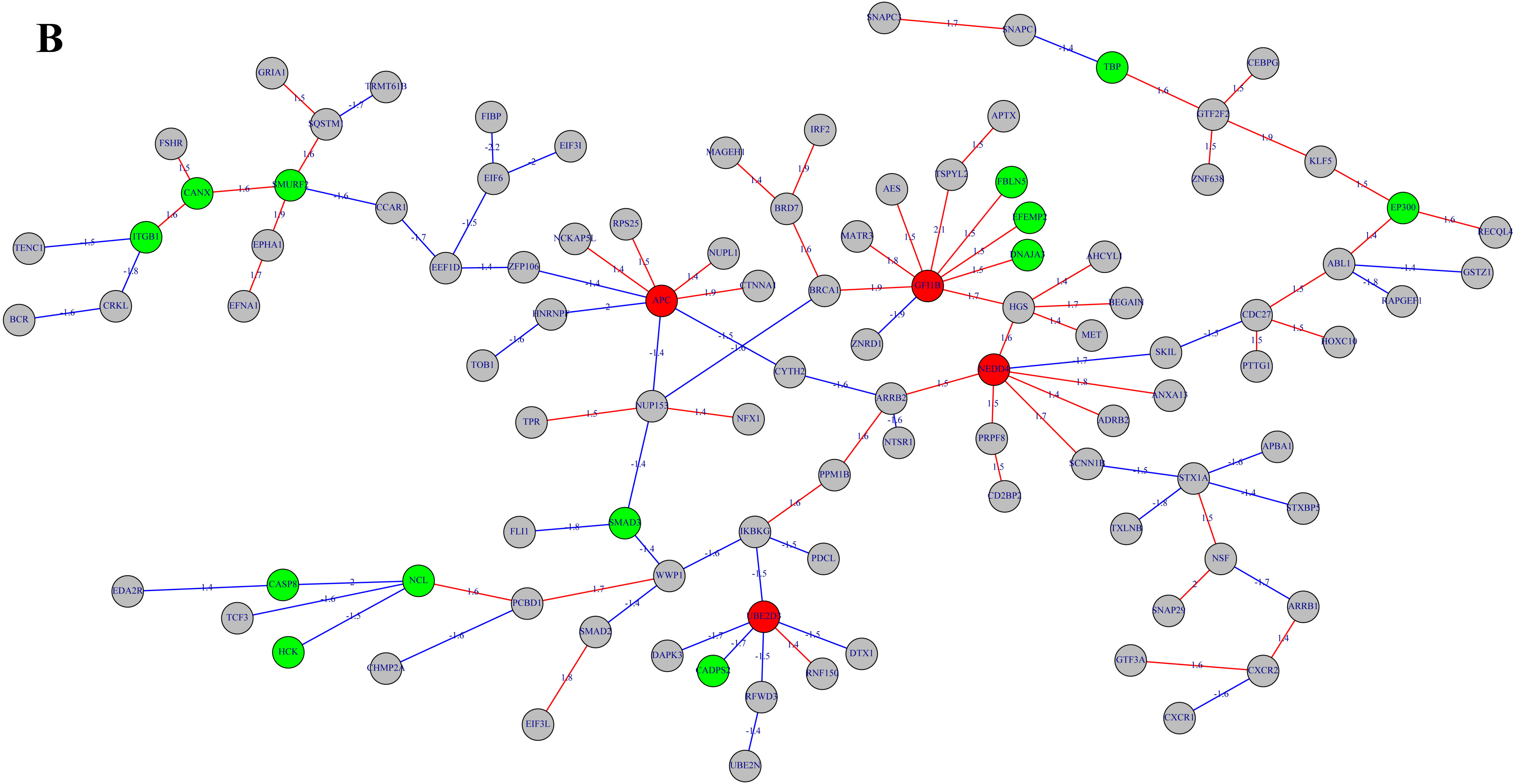
SNAPC3
SNAPC1
TBP
CEBPG
GTF2F2
ZNF638
KLF5
EP300
RECQL4
ABL1
GSTZ1
RAPGEF1
CDC27
HOXC10
PTTG1
SKIL
GRIA1
TRMT61B
SQSTM1
FSHR
CANX
SMURF2
ITGB1
TENC1
CRKL
BCR
EPHA1
EFNA1
CCAR1
FIBP
EIF3I
EIF6
EEF1D
ZFP106
MAGEH1
IRF2
BRD7
APTX
TSPYL2
AES
FBLN5
EFEMP2
DNAJA3
MATR3
GFI1B
ZNRD1
BRCA1
AHCYL1
HGS
BEGAIN
MET
NEDD4
ANXA13
ADRB2
SCNN1B
PRPF8
CD2BP2
ARRB2
NTSR1
CYTH2
NCKAP5L
RPS25
NUPL1
CTNNA1
APC
HNRNPF
TOB1
NUP153
TPR
NFX1
SMAD3
FLI1
WWP1
IKBKG
PDCL
PPM1B
UBE2D3
DAPK3
CADPS2
RFWD3
UBE2N
RNF150
DTX1
NCL
CASP8
EDA2R
TCF3
HCK
PCBD1
CHMP2A
SMAD2
EIF3L
STX1A
APBA1
STXBP5
TXLNB
NSF
SNAP29
ARRB1
GTF3A
CXCR2
CXCR1

C

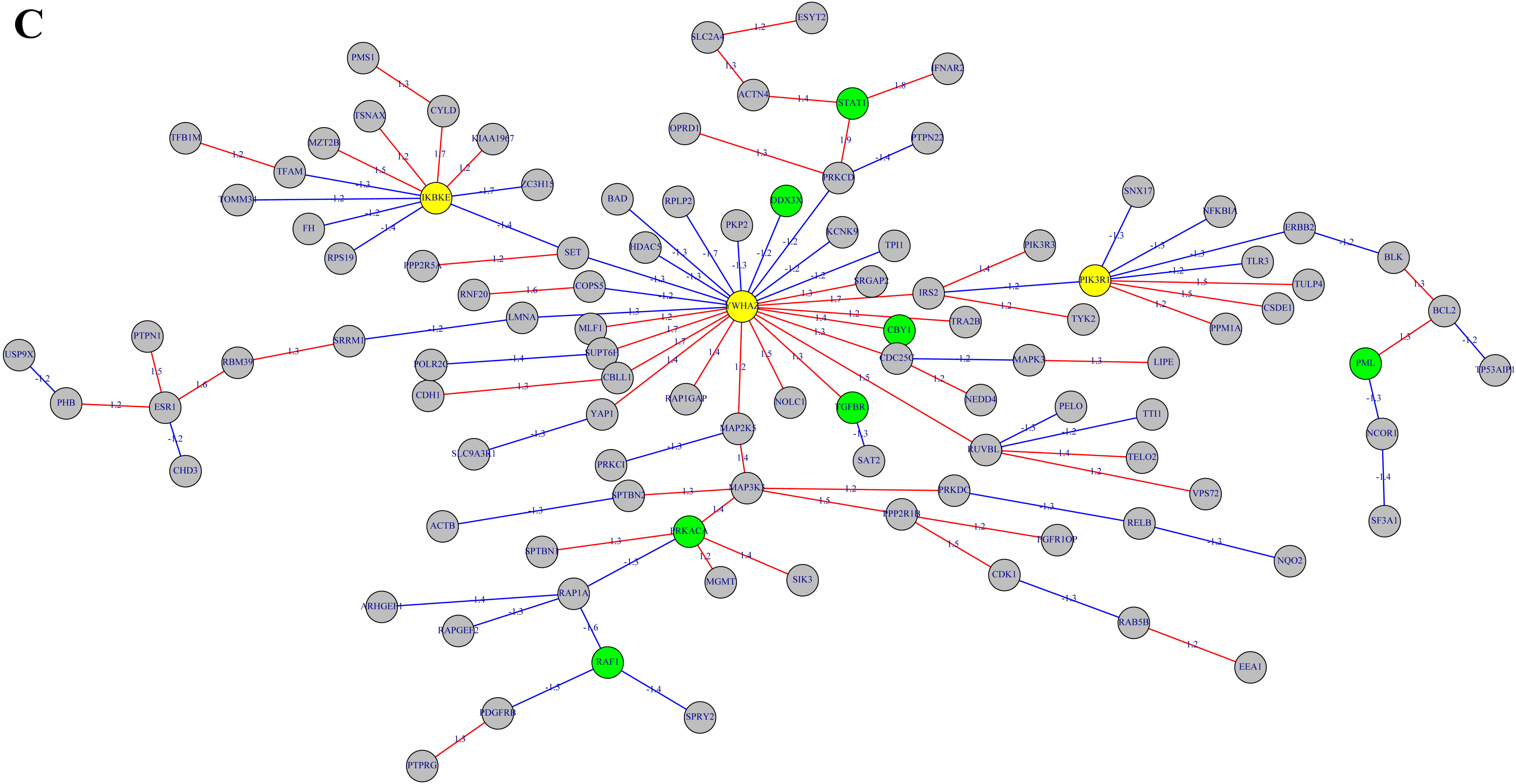
ESYT2
SLC2A4
IFNAR2
PMS1
ACTN4
STAT1
CYLD
TSNAX
OPRD1
PTPN22
TFB1M
MZT2B
KIAA1967
TFAM
PRKCD
ZC3H15
TOMM34
IKBKE
BAD
RPLP2
DDX3X
SNX17
FH
PKP2
NFKBIA
KCNK9
RPS19
HDAC5
ERBB2
SET
TPI1
PIK3R3
PPP2R5A
BLK
TLR3
SRGAP2
PIK3R1
TULP4
COPS5
RNF20
IRS2
CSDE1
YWHAZ
BCL2
LMNA
TRA2B
TYK2
MLF1
CBY1
PPM1A
PTPN1
SRRM1
USP9X
SUPT6H
RBM39
CDC25C
MAPK3
LIPE
PML
TP53AIP1
POLR2C
CBLL1
ESR1
PHB
CDH1
RAP1GAP
NOLC1
TGFBR1
NEDD4
PELO
TTI1
YAP1
MAP2K5
NCOR1
CHD3
SLC9A3R1
RUVBL1
TELO2
PRKCI
SAT2
MAP3K3
PRKDC
VPS72
SPTBN2
SF3A1
ACTB
PPP2R1B
RELB
PRKACA
FGFR1OP
SPTBN1
NQO2
MGMT
SIK3
CDK1
RAP1A
ARHGEF1
RAB5B
RAPGEF2
EEA1
RAF1
PDGFRB
SPRY2
PTPRG

D

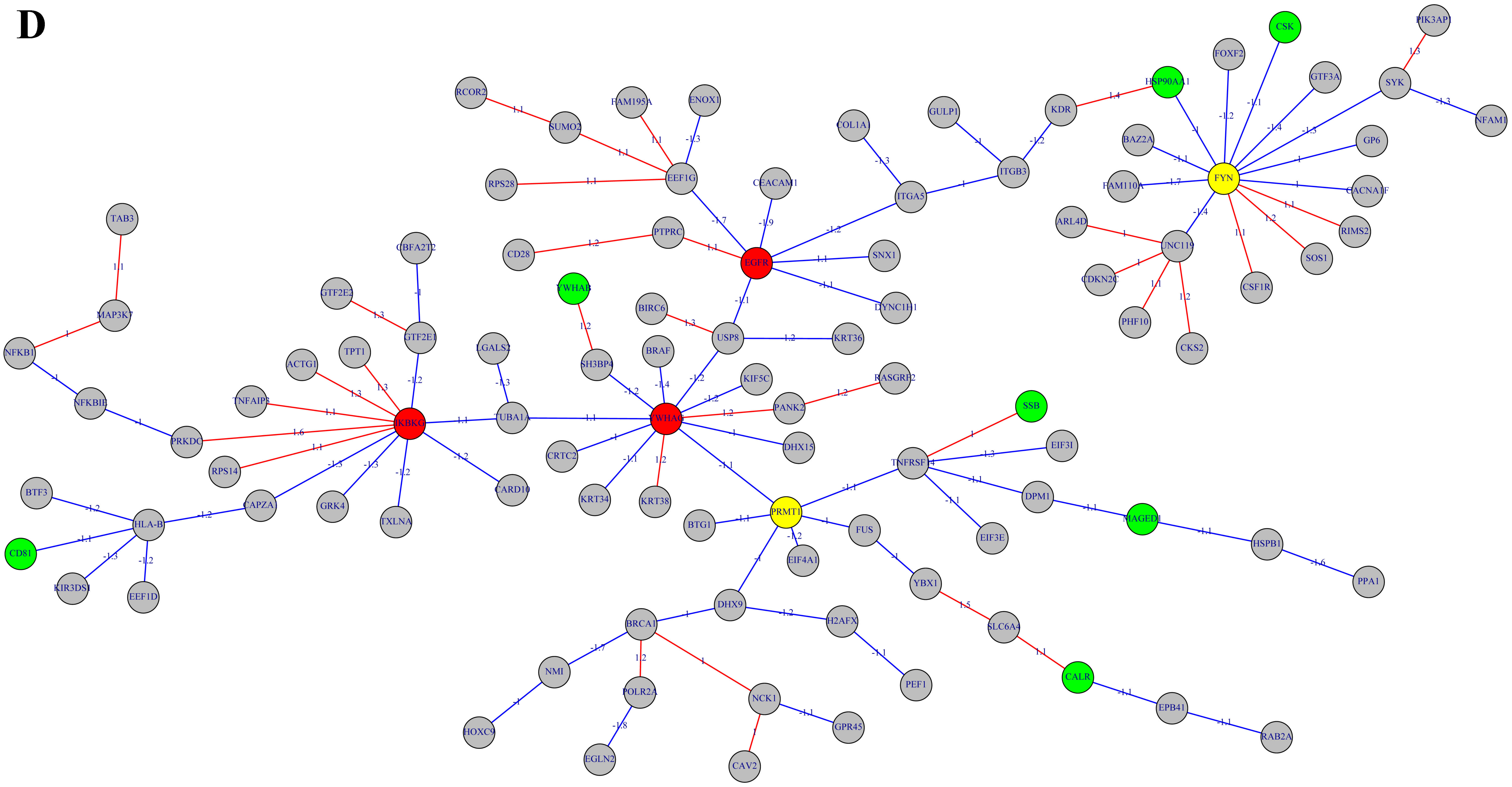

RCOR2
SUMO2
FAM195A
ENOX1
EEF1G
RPS28
CEACAM1
PTPRC
CD28
EGFR
SNX1
DYNC1H1
COL1A1
ITGA5
GULP1
ITGB3
KDR
HSP90AA1
CSK
FOXF2
GTF3A
PIK3AP1
SYK
NFAM1
BAZ2A
GP6
FAM110A
FYN
CACNA1F
ARL4D
UNC119
RIMS2
SOS1
CDKN2C
PHF10
CKS2
CSF1R
TAB3
MAP3K7
NFKB1
NFKBIE
PRKDC
CBFA2T2
GTF2E2
GTF2E1
TPT1
ACTG1
TNFAIP3
IKBKG
RPS14
LGALS2
TUBA1A
CARD10
YWHAB
SH3BP4
BIRC6
USP8
KRT36
BRAF
KIF5C
YWHAG
PANK2
RASGRF2
DHX15
CRTC2
KRT34
KRT38
SSB
EIF3I
TNFRSF14
DPM1
MAGED1
HSPB1
PPA1
EIF3E
PRMT1
BTG1
FUS
EIF4A1
YBX1
SLC6A4
CALR
EPB41
RAB2A
DHX9
H2AFX
PEF1
BRCA1
NMI
HOXC9
POLR2A
EGLN2
NCK1
GPR45
CAV2
BTF3
HLA-B
CAPZA
GRK4
TXLNA
CD81
KIR3DS1
EEF1D
