# Supplementary material for: Dynamic protein interaction modules in human hepatocellular carcinoma progression
Source: BMC Syst Biol. 2013 Dec 9;7(Suppl 5):S2. doi: 10.1186/1752-0509-7-S5-S2 (PMC4029569; doi:10.1186/1752-0509-7-S5-S2)
Supplement: Additional file 5 — Process-wise clusters of dynamic protein interaction modules. This file includes the visual display of the six clusters of process-wise differential co-expression protein interaction modules. [file 1752-0509-7-S5-S2-S5.PDF]

Supplementary Figure 2. Process-wise clusters of dynamic protein interaction modules. Altogether six clusters (Clusters **I**, **II**, **III**, **IV**, **V**, and **VI**) were obtained. the connected PPIs in one cluster are functionally related (because of PPI connection) and have similar expression correlation dynamics in HCC development. Node color differentiates different node types: green for HCV-protein-binding proteins, red for hub nodes (degree ≥ 5), and yellow for both HCV-binding protein and hub.

Cluster I

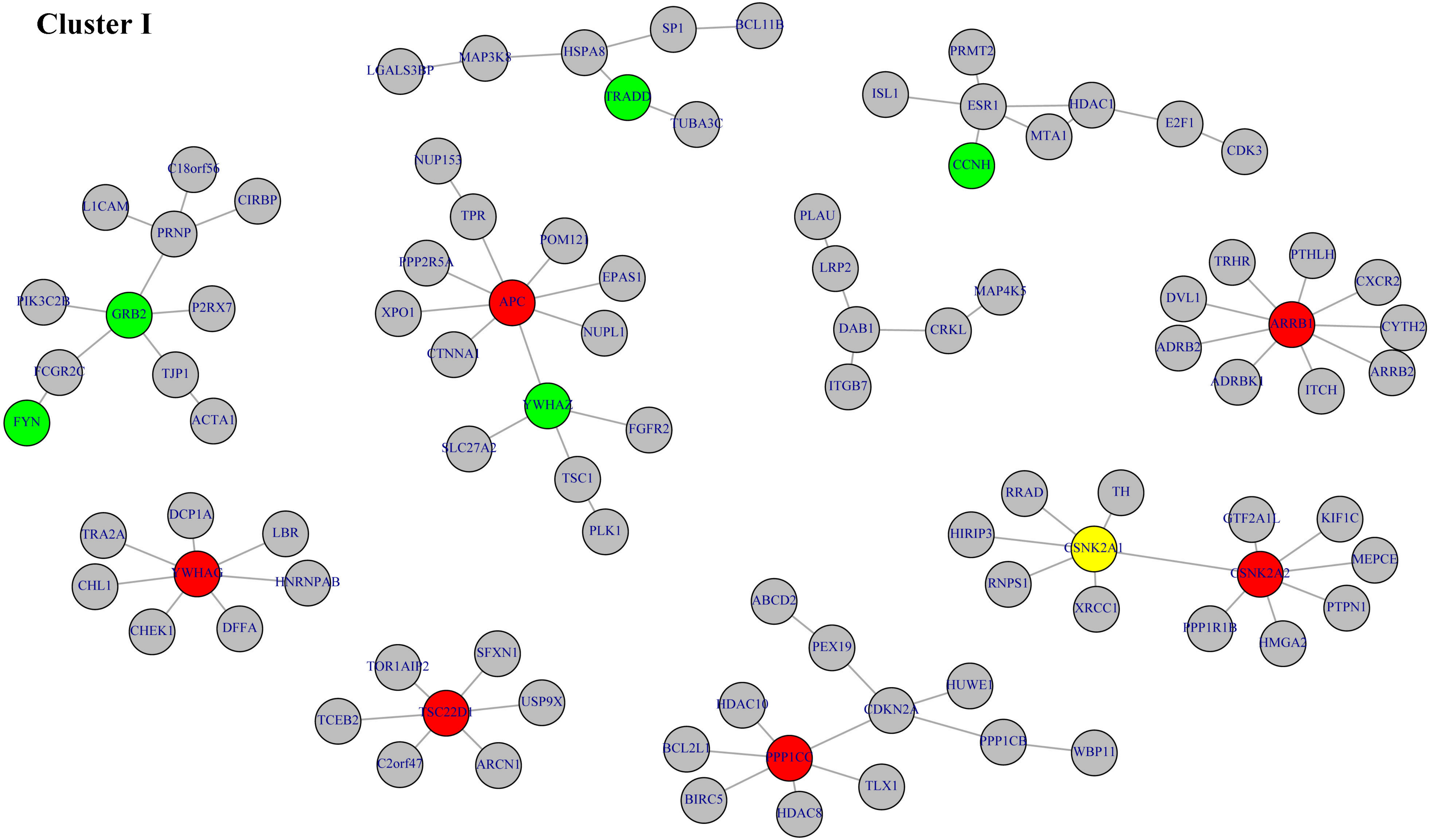
LGALS3BP
MAP3K8
HSPA8
SP1
BCL11B
TRADD
TUBA3C
PRMT2
ISL1
ESR1
HDAC1
E2F1
CDK3
MTA1
CCNH
NUP153
TPR
POM121
PPP2R5A
EPAS1
XPO1
APC
NUPL1
CTNNA1
YWHAZ
FGFR2
SLC27A2
TSC1
PLK1
C18orf56
L1CAM
CIRBP
PRNP
PIK3C2B
GRB2
P2RX7
FCGR2C
TJP1
FYN
ACTA1
PLAU
LRP2
DAB1
CRKL
MAP4K5
ITGB7
TRHR
PTHLH
CXCR2
DVL1
ARRB1
CYTH2
ADRB2
ADRBK1
ITCH
ARRB2
DCP1A
TRA2A
LBR
CHL1
YWHAG
HNRNPAB
CHEK1
DFFA
RRAD
TH
HIRIP3
CSNK2A1
RNPS1
XRCC1
GTF2A1L
KIF1C
MEPCE
CSNK2A2
PTPN1
PPP1R1B
HMGA2
ABCD2
PEX19
TOR1AIP2
SFXN1
USP9X
TCEB2
TSC22D1
C2orf47
ARCN1
HUWE1
CDKN2A
HDAC10
PPP1CB
WBP11
BCL2L1
PPP1CC
TLX1
BIRC5
HDAC8

Cluster II

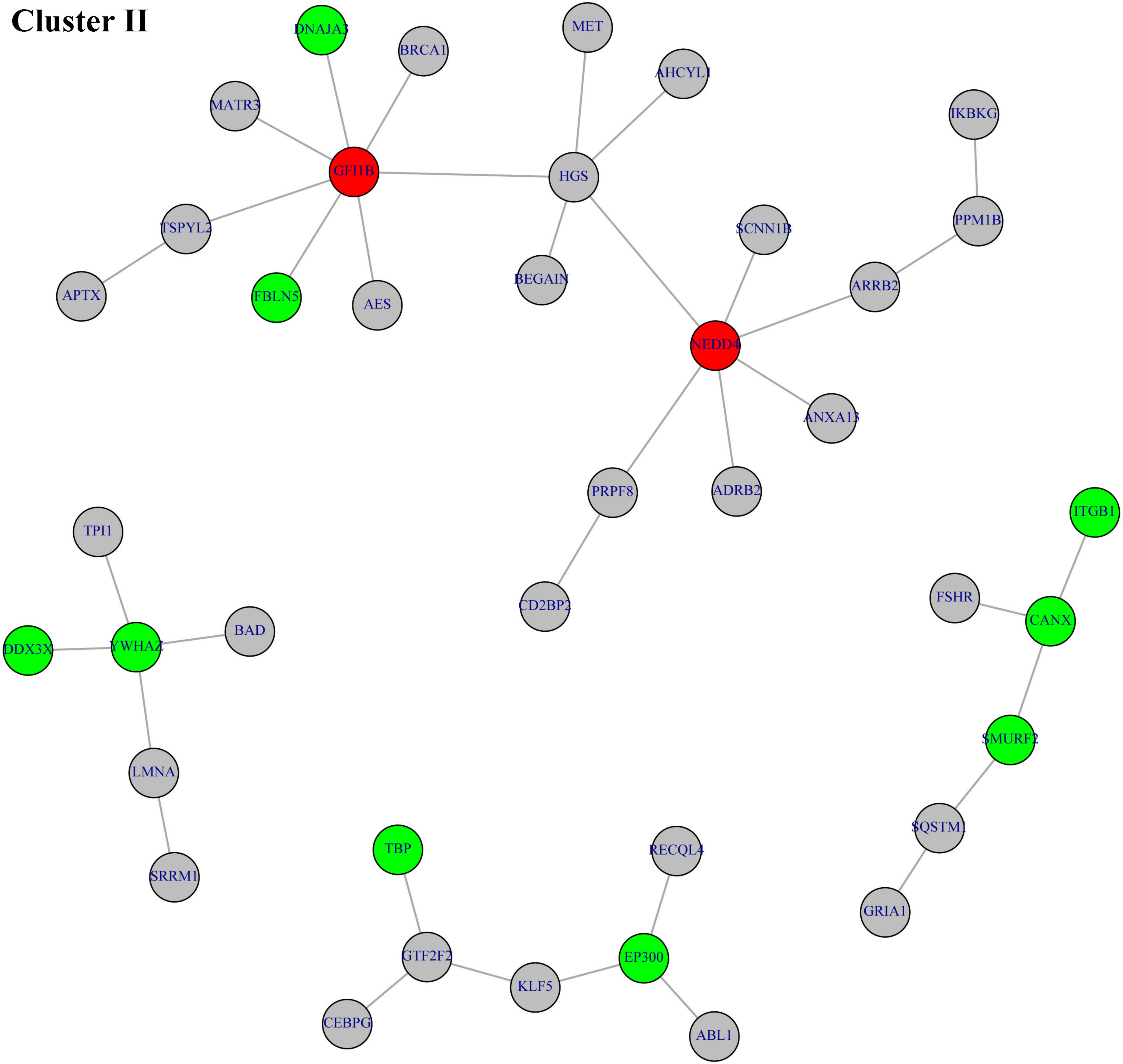
DNAJA3
BRCA1
MET
AHCYL1
IKBKG
MATR3
GFI1B
HGS
TSPYL2
PPM1B
SCNN1B
APTX
FBLN5
AES
BEGAIN
ARRB2
NEDD4
ANXA13
PRPF8
ADRB2
TPI1
ITGB1
CD2BP2
FSHR
CANX
BAD
DDX3X
YWHAZ
SMURF2
LMNA
SQSTM1
TBP
RECQL4
SRRM1
GRIA1
GTF2F2
EP300
KLF5
CEBPG
ABL1

Cluster III

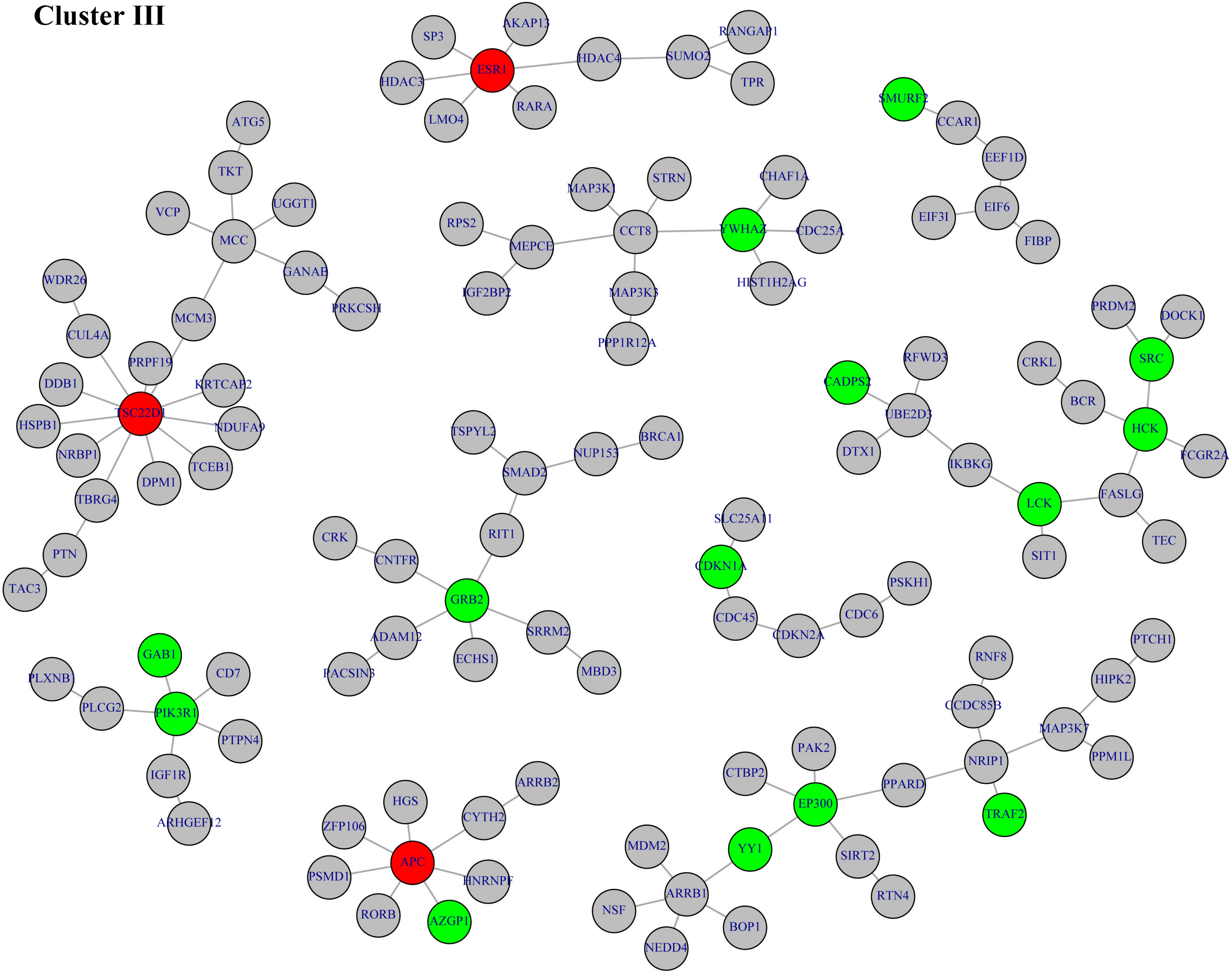

AKAP13
SP3
ESR1
HDAC3
LMO4
RARA
HDAC4
SUMO2
RANGAP1
TPR
SMURF2
CCAR1
EEF1D
EIF6
EIF3I
FIBP
ATG5
TKT
VCP
UGGT1
MCC
GANAB
PRKCSH
MCM3
WDR26
CUL4A
PRPF19
DDB1
KRTCAP2
TSC22D1
HSPB1
NDUFA9
NRBP1
TCEB1
DPM1
TBRG4
PTN
TAC3
RPS2
MEPCE
IGF2BP2
MAP3K1
STRN
CCT8
MAP3K3
PPP1R12A
CHAF1A
YWHAZ
CDC25A
HIST1H2AG
RFWD3
CADPS2
UBE2D3
DTX1
IKBKG
PRDM2
DOCK1
SRC
CRKL
BCR
HCK
FCGR2A
FASLG
LCK
SIT1
TEC
TSPYL2
SMAD2
NUP153
BRCA1
RIT1
CRK
CNTFR
GRB2
ADAM12
PACSIN3
ECHS1
SRRM2
MBD3
SLC25A11
CDKN1A
CDC45
CDKN2A
CDC6
PSKH1
GAB1
CD7
PLXNB1
PLCG2
PIK3R1
PTPN4
IGF1R
ARHGEF12
RNF8
PTCH1
HIPK2
CCDC85B
MAP3K7
PPM1L
NRIP1
TRAF2
PAK2
CTBP2
PPARD
EP300
SIRT2
RTN4
YY1
MDM2
ARRB1
NSF
BOP1
NEDD4
HGS
ZFP106
ARRB2
CYTH2
APC
PSMD1
HNRNPF
RORB
AZGP1

Cluster IV

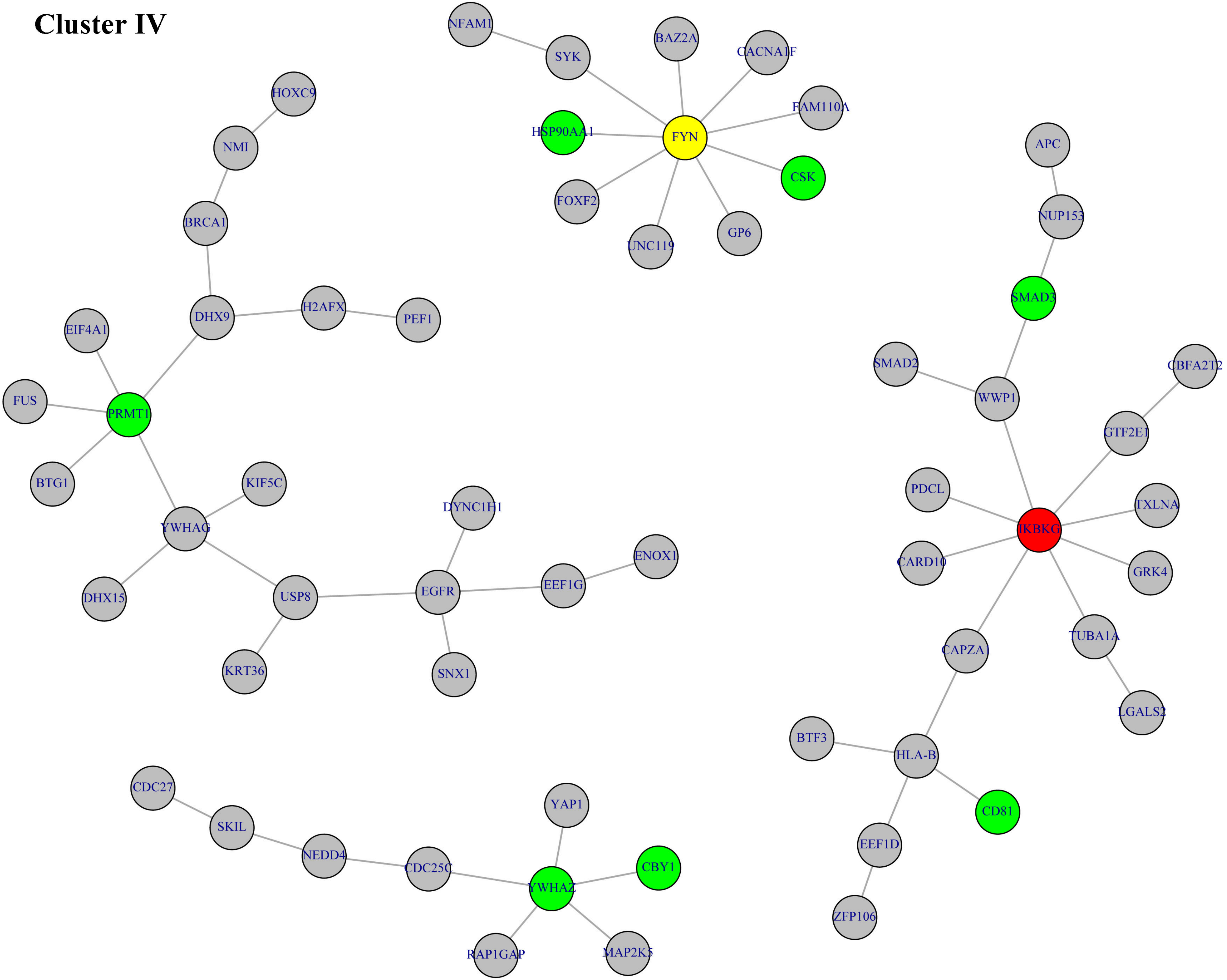
NFAM1
SYK
BAZ2A
CACNA1F
FAM110A
HSP90AA1
FYN
CSK
FOXF2
UNC119
GP6
HOXC9
NMI
BRCA1
DHX9
H2AFX
PEF1
EIF4A1
FUS
PRMT1
BTG1
KIF5C
YWHAG
DHX15
USP8
KRT36
DYNC1H1
EGFR
EEF1G
ENOX1
SNX1
APC
NUP153
SMAD3
SMAD2
WWP1
CBFA2T2
GTF2E1
PDCL
TXLNA
IKBKG
CARD10
GRK4
TUBA1A
CAPZA1
LGALS2
BTF3
HLA-B
CD81
EEF1D
ZFP106
CDC27
SKIL
NEDD4
CDC25C
YAP1
YWHAZ
CBY1
RAP1GAP
MAP2K5

Cluster V

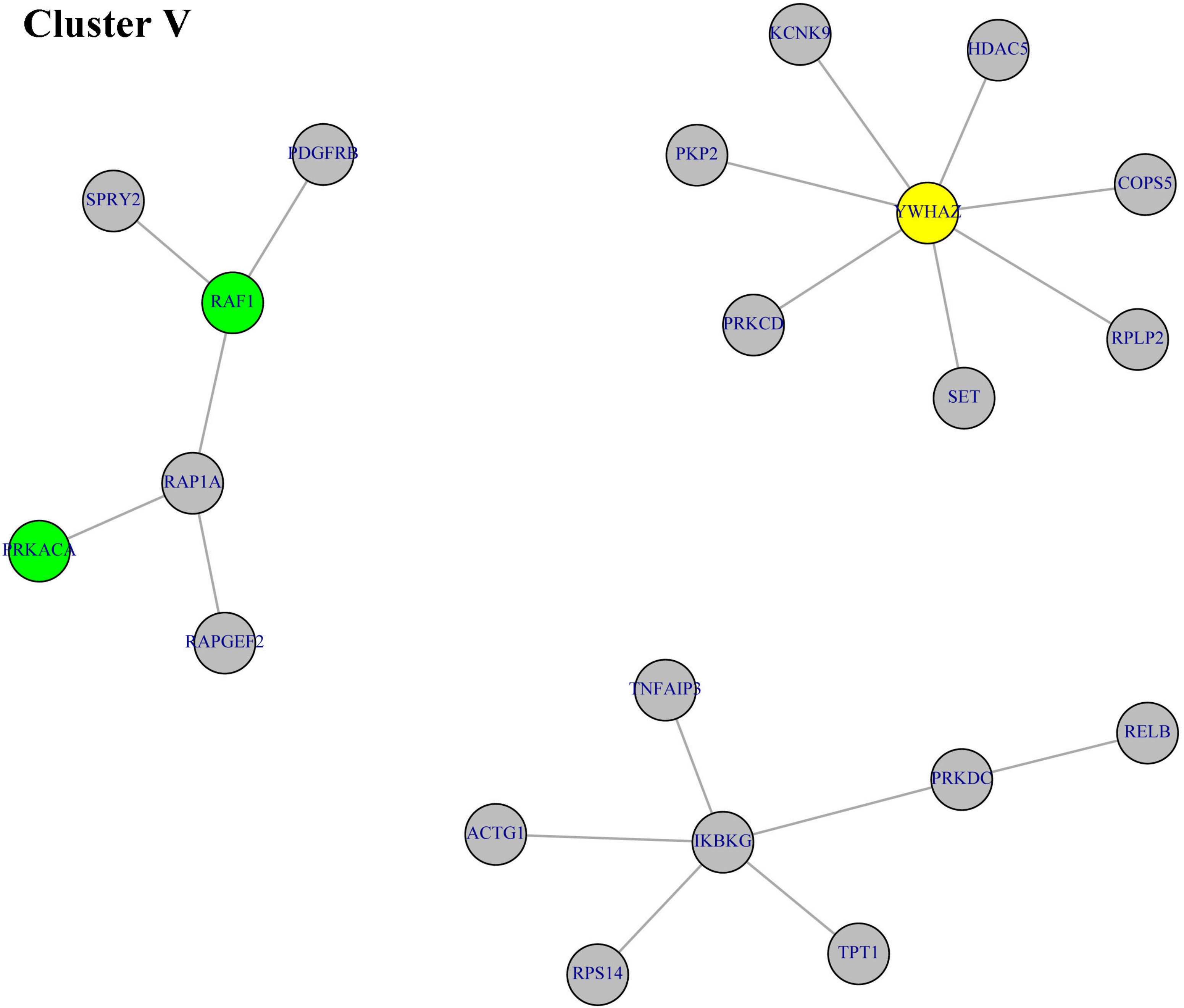

PDGFRB
SPRY2
RAF1
RAP1A
PRKACA
RAPGEF2
KCNK9
HDAC5
PKP2
YWHAZ
COPS5
PRKCD
RPLP2
SET
TNFAIP3
RELB
PRKDC
ACTG1
IKBKG
TPT1
RPS14

Cluster VI

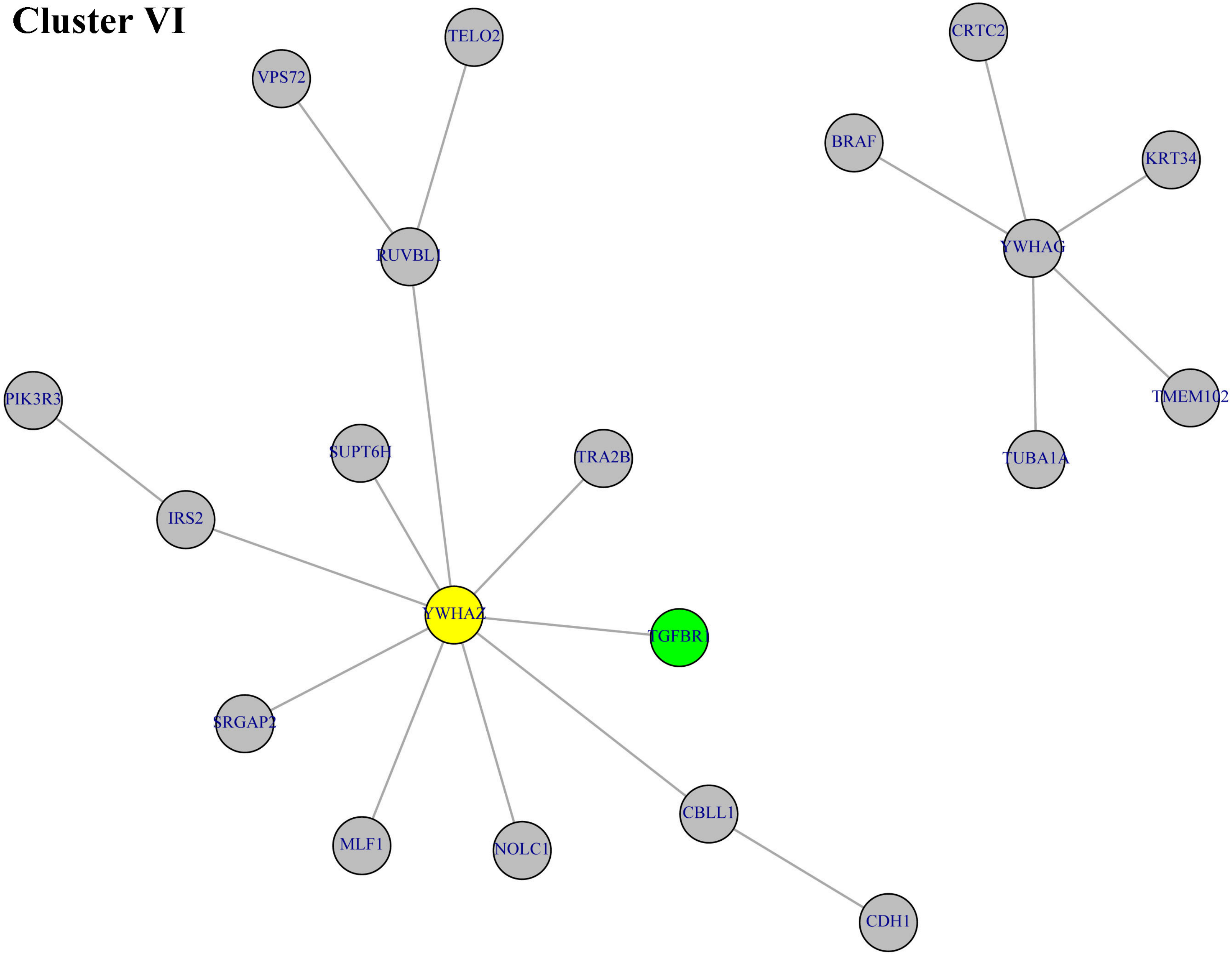

TELO2
VPS72
RUVBL1
CRTC2
BRAF
KRT34
YWHAG
TMEM102
TUBA1A
PIK3R3
SUPT6H
TRA2B
IRS2
YWHAZ
TGFBR1
SRGAP2
CBLL1
MLF1
NOLC1
CDH1
